# Intravenous and Oral Fluid Therapy in Neonatal Calves With Diarrhea or Sepsis and in Adult Cattle

**DOI:** 10.3389/fvets.2020.603358

**Published:** 2021-01-27

**Authors:** Peter D. Constable, Florian M. Trefz, Ismail Sen, Joachim Berchtold, Mohammad Nouri, Geoffrey Smith, Walter Grünberg

**Affiliations:** ^1^Department of Veterinary Clinical Medicine, College of Veterinary Medicine, University of Illinois at Urbana-Champaign, Urbana, IL, United States; ^2^Clinic for Ruminants, Vetsuisse Faculty, University of Bern, Bern, Switzerland; ^3^Department of Internal Medicine, Faculty of Veterinary Medicine, Kyrgyz-Turkish Manas University, Bishkek, Kyrgyzstan; ^4^Tierärztliche Gemeinschaftspraxis Dr. Berchtold & Dr. Taschke, Pittenhart, Germany; ^5^Department of Clinical Sciences, Faculty of Veterinary Medicine, Shahid Chamran University of Ahvaz, Ahvaz, Iran; ^6^Department of Population Health & Pathobiology, College of Veterinary Medicine, North Carolina State University, Raleigh, NC, United States; ^7^Foundation, Clinic for Cattle, University of Veterinary Medicine Hannover, Hannover, Germany

**Keywords:** osmolality, alkalosis, acidosis, fluid therapy, acid–base balance, oral electrolyte solution

## Abstract

Optimal fluid therapy protocols in neonatal calves and adult cattle are based on consideration of signalment, history, and physical examination findings, and individually tailored whenever laboratory analysis is available. Measurement of the magnitude of eye recession, duration of skin tenting in the lateral neck region, and urine specific gravity by refractometry provide the best estimates of hydration status in calves and cattle. Intravenous and oral electrolyte solutions (OES) are frequently administered to critically ill calves and adult cattle. Application of physicochemical principles indicates that 0.9% NaCl, Ringer's solution, and 5% dextrose are equally acidifying, lactated Ringer's and acetated Ringer's solution are neutral to mildly acidifying, and 1.3–1.4% sodium bicarbonate solutions are strongly alkalinizing in cattle. Four different crystalloid solutions are recommended for intravenous fluid therapy in dehydrated or septic calves and dehydrated adult cattle: (1) lactated Ringer's solution and acetated Ringer's solution for dehydrated calves, although neither solution is optimized for administration to neonatal calves or adult cattle; (2) isotonic (1.3%) or hypertonic (5.0 or 8.4%) solutions of sodium bicarbonate for the treatment of calves with diarrhea and severe strong ion (metabolic) acidosis and hyponatremia, and adult cattle with acute ruminal acidosis; (3) Ringer's solution for the treatment of metabolic alkalosis in dehydrated adult cattle, particularly lactating dairy cattle; and (4) hypertonic NaCl solutions (7.2%) and an oral electrolyte solution or water load for the rapid resuscitation of dehydrated neonatal calves and adult cattle. Much progress has been made since the 1970's in identifying important attributes of an OES for diarrheic calves. Important components of an OES for neonatal calves are osmolality, sodium concentration, the effective SID that reflects the concentration of alkalinizing agents, and the energy content. The last three factors are intimately tied to the OES osmolality and the abomasal emptying rate, and therefore the rate of sodium delivery to the small intestine and ultimately the rate of resuscitation. An important need in fluid and electrolyte therapy for adult ruminants is formulation of a practical, effective, and inexpensive OES.

## Introduction

Intravenous solution formulations should be optimized for different species (1) and fluid therapy should be patient-centered and not follow a “one fluid for all” principle (2). Five abnormalities can concurrently exist in critically ill neonatal calves and adult cattle: (a) free water deficit; (b) abnormal plasma electrolyte concentrations; (c) acid–base abnormalities; (d) plasma osmolality abnormalities; and (e) plasma oncotic pressure imbalances. Optimal fluid therapy protocols in neonatal calves and adult cattle are based on consideration of signalment, history, and physical examination findings, and should be individually tailored if laboratory analysis is available. The signalment, history, and clinical diagnosis will suggest the likely presence of decreased free water volume and abnormalities in plasma electrolyte concentrations, acid-base balance, osmolality and oncotic pressure. Serum biochemical analysis, particularly measurement of the plasma total CO_2_, chloride, sodium, potassium, and calcium concentrations is recommended to optimize fluid therapy in critically ill adult cattle, with the addition of acid-base analysis in critically ill neonatal calves. Unfortunately, most critically ill neonatal calves and adult cattle are treated in resource poor environments on the farm without ready access to laboratory analysis. In these circumstances the clinician determines the nature and degree of the abnormalities likely to be present based on the animal's clinical presentation (3) and uses the information to select the most appropriate intravenous fluid formulation for treatment.

**Figure 1:**
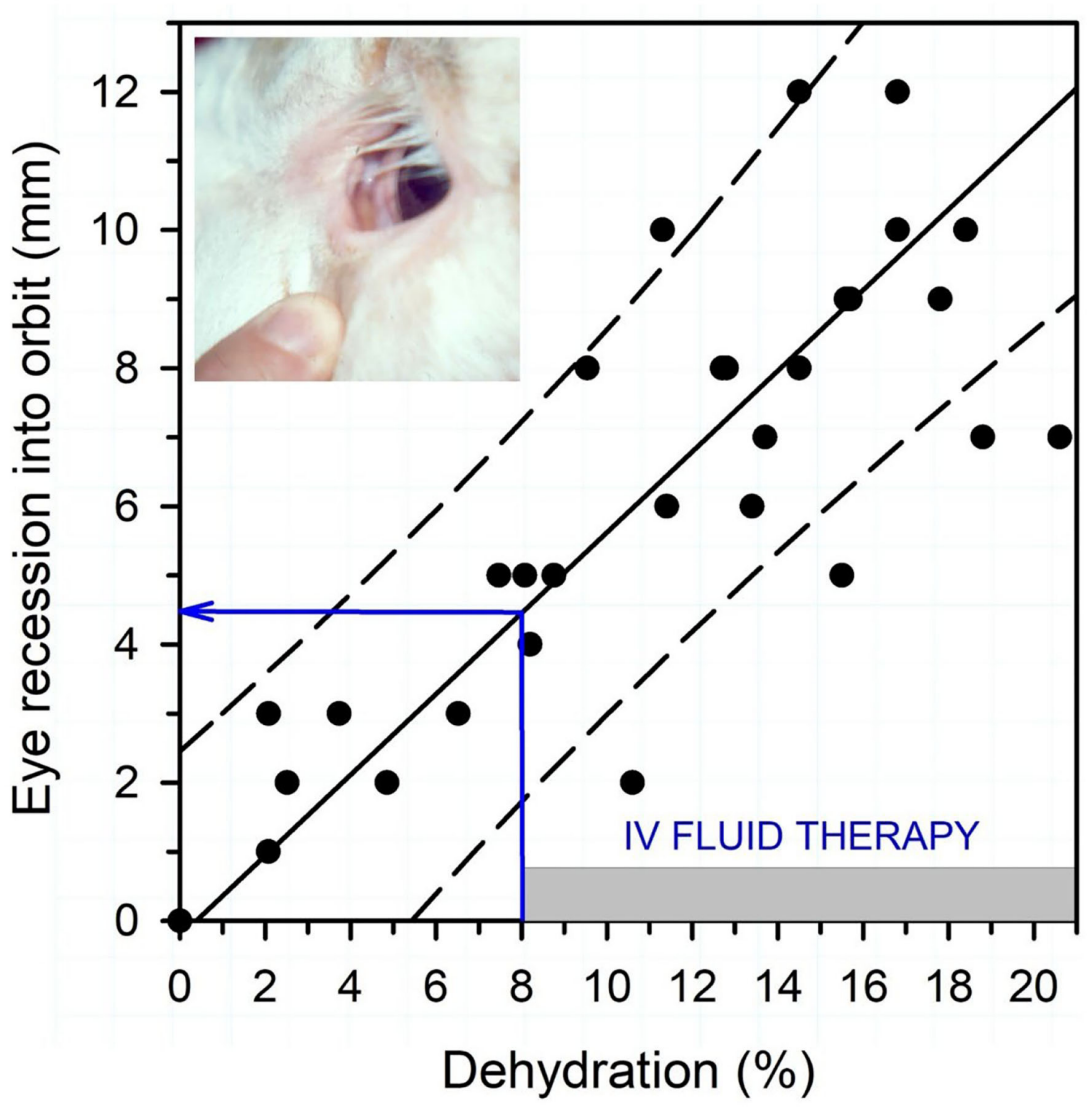
Association between eye recession into the orbit and dehydration (percent body weight) in 15 neonatal calves with experimentally induced diarrhea and dehydration. The filled circles are individual data points (total of 45, 3/calf), the solid line is the linear regression line, and the dashed lines are the 95% confidence interval for prediction. Dehydration (percent of body weight) can be estimated by multiplying the degree of eye recession into the orbit in mm by 1.6. Intravenous fluid administration is recommended when dehydration is estimated at 8% or more of body weight, equivalent to an eye recession into the orbit of 4 mm or more. Figure modified with permission from: Constable et al. (4).

**Figure 2:**
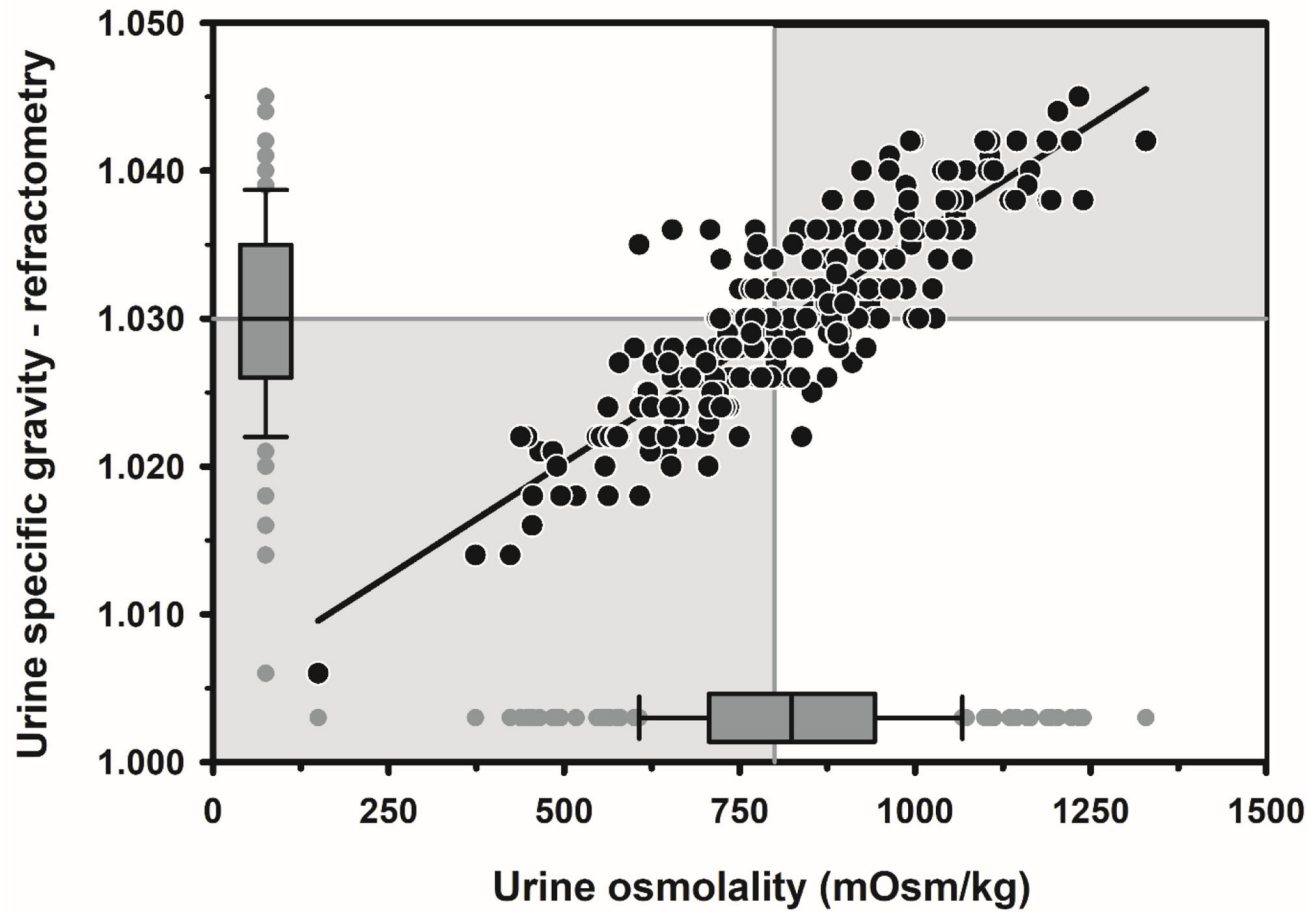
Scatterplot of the linear relationship between urine specific gravity measured by optical refractometry (USG-R) and urine osmolality (UOsm, reference method) for 242 urine samples obtained periodically from 20 multiparous periparturient Holstein-Friesian cows from late gestation to early lactation. Some data points are superimposed. The solid black line is the linear regression line. The solid gray vertical line indicates the recommended threshold value for diagnosing dehydration (UOsm ≥ 800 mOsm/kg), and the solid gray horizontal line indicates the optimal cut point of USG-R (≥ 1.030) identified by logistic regression for diagnosing dehydration. The box and whiskers plots represent the median (middle line), interquartile range (ends of the shaded rectangle), 10% to 90% confidence interval (whiskers), and values outside this confidence interval (small gray circles). Figure modified with permission from: Megahed et al. (5).

**Figure 3:**
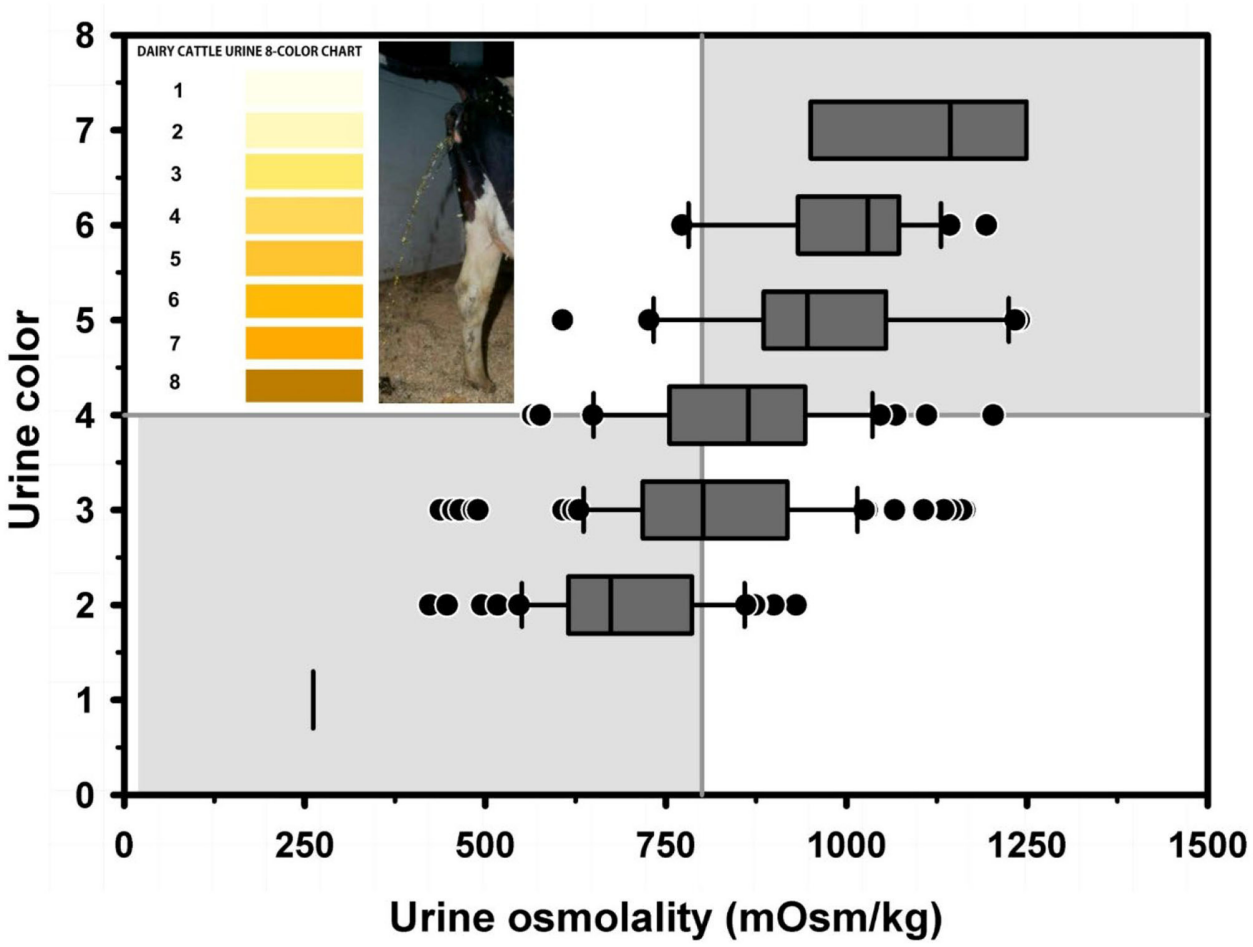
Box and whiskers plot of the association between urine color (8 levels) and urine osmolality (UOsm; reference method) for 237 urine samples obtained periodically from 20 multiparous periparturient Holstein-Friesian cows. The solid gray vertical line indicates the recommended cut point for UOsm (≥800 mOsm/kg) for diagnosing dehydration. The solid gray horizontal line indicates the optimal cut point for color (≥4) identified by logistic regression for diagnosing dehydration. See Figure 2 legend for additional information. Figure modified with permission from: Megahed et al. (5).

**Figure 4:**
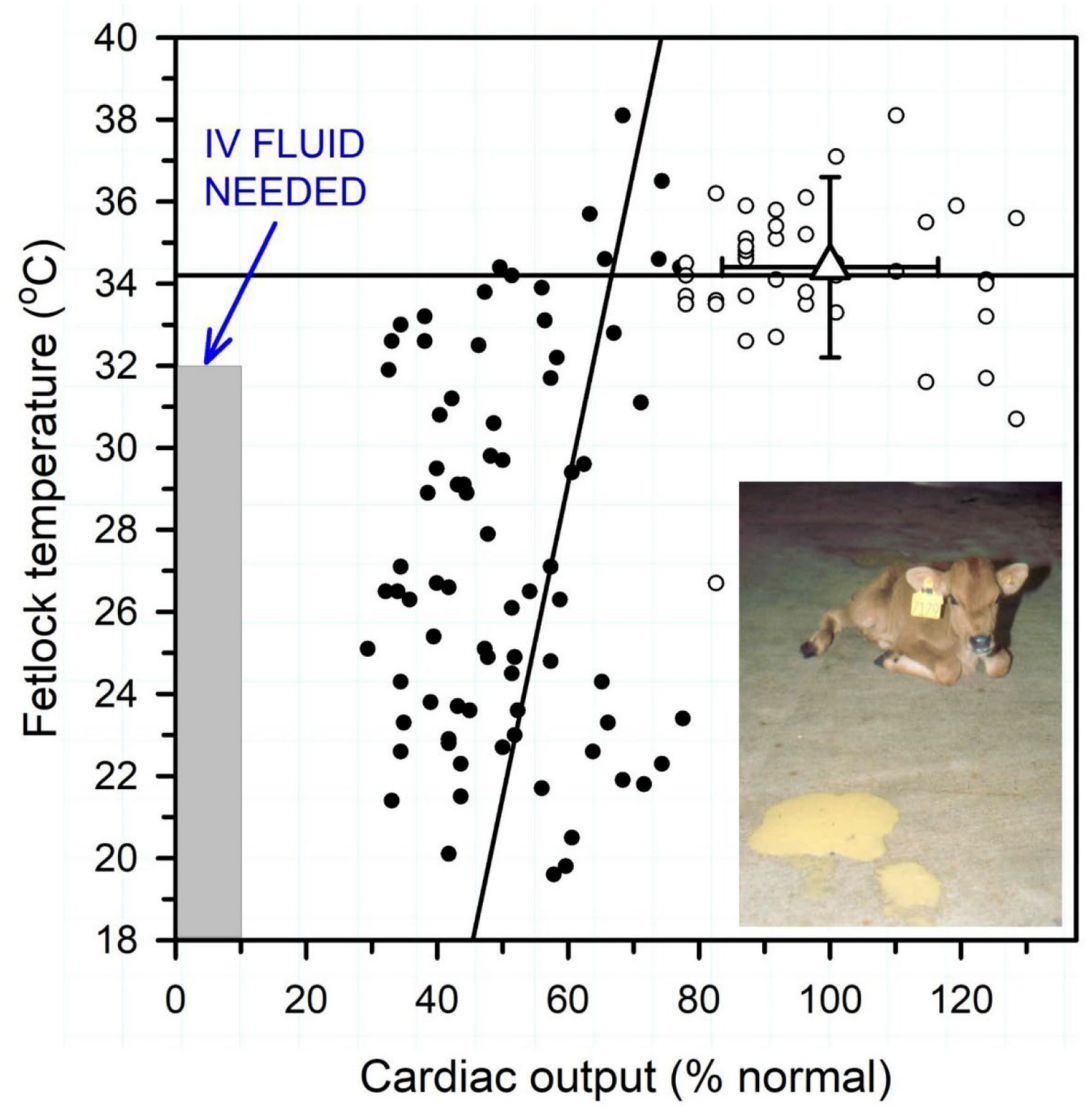
Relationship between peripheral temperature (hind fetlock) and cardiac output (percent of normal) in diarrheic calves with varying degrees of dehydration housed in a thermoneutral environment. Normal mean cardiac output was 8.5 L/min or 218 ml/min/kg BW. Linear regression lines for cardiac output < 65% normal (solid circles) and >65% normal (open circles). Triangle with error bars indicates reference values for healthy neonatal calves. Note that fetlock temperature is positively correlated with cardiac output when cardiac output <65% of normal. Intravenous fluid therapy is recommended when hindlimb fetlock temperature is <32°C (<90°F) when calves are housed in a thermoneutral environment. Figure modified with permission from Constable et al. (6).

**Figure 5:**
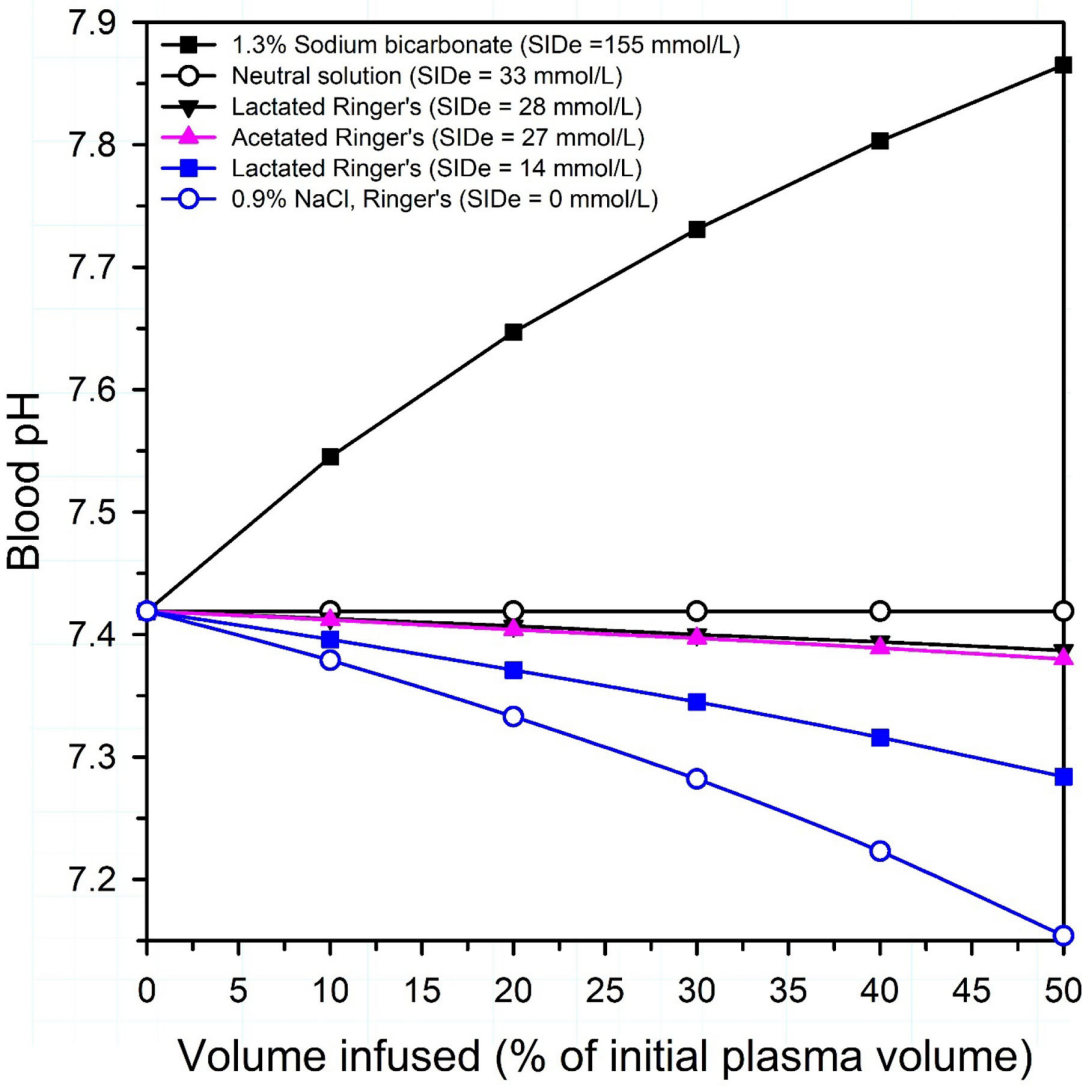
Theoretical effect of intravenous administration of four crystalloid solutions on venous blood pH in healthy neonatal calves. SIDe = the effective strong ion difference between the charge of strong cations (Na in 1.3% sodium bicarbonate and 0.9% NaCl; Na, K and Ca in lactated Ringer's, Na, K, and Mg in acetated Ringer's) and strong anions that are not metabolized or are minimally metabolized by the calf (Cl in 0.9% NaCl; Cl and D-Lactate in lactated Ringer's; Cl and gluconate in acetated Ringer's). Because D-lactate in lactated Ringer's is present in a racemic mixture in some commercially available formulations, lactated Ringer's is presented as two solutions: (1) an equimolar solution of L-lactate and D-lactate, with an SIDe = 14 mmol/L; (2) a solution where lactate is only in the L-isomer form, with an SIDe = 28 mmol/L. Note that all solutions, except 1.3% sodium bicarbonate, are acidifying in neonatal calves. Adapted from: Constable (19).

Textbook dogma has long promulgated the belief that only moderate to marked dehydration can be clinically detected in domestic animals. Experimental studies have demonstrated that mild dehydration can be readily detected in neonatal calves, in that a dehydration of 2% body weight is associated with slight enophthalmos of 1 mm, dry mucous membranes, and a prolonged skin tent duration to 3 s (4). Dehydration (percent body weight) can be accurately quantified in neonatal calves by multiplying the degree of eye recession into the orbit in mm by 1.6 [Figure 1; (4)]. It is not known whether a similar relationship exists for adult cattle. Measurement of urine specific gravity by refractometry is also clinically helpful in assessing urine osmolality and therefore free water balance [Figure 2; (5)]. Urine color has moderate clinical utility for assessing urine osmolality in cattle (Figure 3), but measurement of urine specific gravity by dipstick is not recommended because it provides an inaccurate measure of urine osmolality (5).

Reductions in cardiac output in dehydrated calves are associated with decreased peripheral perfusion and therefore cooler extremities. Peripheral (fetlock) temperature, and the related core-peripheral temperature difference, can therefore be used to guide the need for intravenous fluid therapy when measured in temperature-controlled surroundings (6). The clinical utility of peripheral temperature as a predictor of the need for intravenous fluid therapy was first investigated in critically ill humans in the 1950's, when it was observed that the prognosis for survival was decreased as the temperature of the big toe approached the ambient room temperature. A portable low cost hand-held infrared thermographic unit provides a quantitative tool to measure the peripheral temperature, particularly the hind fetlocks, of neonatal calves with diarrhea (6). A core-peripheral temperature difference of >13°F (>7°C) in neonatal calves with diarrhea indicated that cardiac output was <65% of the mean value for healthy calves, and after this point hindlimb fetlock temperature decreased with additional reductions in cardiac output (Figure 4). Peripheral temperature can also be estimated by using the hand to palpate the hind fetlocks or ears and the perceived temperature compared to the peripheral temperature of healthy calves housed in the same environment (6).

Primary therapeutic objectives of fluid therapy are to correct existing abnormalities and to provide maintenance therapy. Existing abnormalities typically require 4–6 h to correct and maintenance therapy may be needed for 2–4 days (7). The estimated volume required to rehydrate the animal and correct electrolyte and acid-base abnormalities should be given intravenously in the first 4–6 h. The normalization of circulating blood volume will increase renal blood flow and glomerular filtration rate and thereby restore renal function and facilitate the correction of electrolyte and acid–base abnormalities. Other routes of parenteral fluid administration (subcutaneous, intraperitoneal) are inferior to the intravenous route for the rapid resuscitation of critically ill neonatal calves (8) and adult cattle.

The amount of fluid required and route of administration depends on the initial degree of dehydration, and an estimate of the continuous losses that occur during treatment, in conjunction with the animal's maintenance requirements during treatment due to minimal intake of water, electrolytes and nutrients. The total volume of fluid to correct dehydration is calculated based on percent dehydration and body weight (BW). In animals that are sufficiently dehydrated to warrant intravenous fluid therapy, at least 3 to 5 L of intravenous fluid is required in a neonatal calf (8% dehydrated × 50 kg BW), and at least 30–50 L in an adult cow (8% dehydrated × 650 kg BW). As previously mentioned, there are two stages to fluid administration: (1) intravenous hydration/corrective therapy in the first 4–6 h (total volume of 100–150 mL/kg BW); and (2) intravenous or oral maintenance therapy in the following 20–24 h (total volume of 60–150 ml/kg BW/24 h) (8). Practical methods for the placement and maintenance of intravenous catheters (auricular vein, jugular vein) in neonatal calves and adult cattle, techniques for administration of intravenous and oral fluids, and the importance of solution temperature in neonatal calves are described in detail elsewhere (8–11). Intravenous fluids should be warmed before administration as large volume administration of fluids <38°C will cool the calf. However, it must be recognized that even when prewarmed fluids (38°C) are administered that the fluid temperature is decreased below core body temperature when it reaches the calf because solution heat is lost to the environment (12). Fluid warming devices placed around the fluid administration line are efficacious depending on the flow rate, but only when placed as close to the animal as possible (12), which can be challenging in some calves.

Neonatal calves and adult cattle should be monitored during the first hour of intravenous hydration/corrective therapy for evidence of clinical improvement or the development of deleterious effects. The fastest intravenous administration rate of isotonic solutions for resuscitation of critically ill calves in the first hour is 80 ml/kg BW over the first hour of infusion (13), although 50 ml/kg BW has been recommended to minimize the risk of hyperhydration (14). A favorable response in neonatal calves is indicated by an improvement in mental attitude and hydration status within 30–60 min, followed by urination in calves that can stand. Monitoring mean central venous pressure is not practical as sufficiently long catheters are commercially unavailable and technically difficult to place, and because of the large between animal variability in measured central venous pressures (15). Unfavorable responses to treatment include development of a moist cough and tachypnea due to pulmonary edema formation because of a too rapid rate of fluid administration or pre-existing pneumonia and failure to urinate because of acute renal failure. Intravenous fluid administration should be stopped when these clinical signs are observed. The response to intravenous hydration/corrective therapy is slower in adult cattle because fluid administration rates rarely exceed 30 ml/kg BW per hour due to physical constraints related to catheter size and height of the fluid reservoir above the heart (11). Typical intravenous fluid administration rates of 20–30 ml/kg per hour have been recommend for intravenous hydration/corrective therapy in adult cattle (11, 16).

### Strong Ion Difference of Plasma, Intravenous Fluids, and Oral Electrolyte Solutions

Fluids can contain charged substances that are positively or negatively charged. Charged substances may have a charge that is fixed and unaltered under physiologic conditions in biological systems, in which case the substances are called strong cations if positively charged, and strong anions if negatively charged. Alternatively, charged substances may have a charge that is variable and dependent on pH under physiologic conditions in biological systems, in which case the substances are called buffer ions, with a net charge varying from positive to zero to negative, depending on the solution pH. Electroneutrality must be preserved; consequently, the sum of the strong cations and positive buffer ions must equal the sum of strong anions and negative buffer ions at a specific pH (17–19).

The strong ion difference (SID) represents the difference between the charge assigned to quantitatively important strong cations (Na^+^, K^+^, Ca^2+^, Mg^2+^) and strong anions (Cl^−^, L-lactate^−^, D-lactate^−^, sulfate^2−^, ketoacids, non-esterified fatty acids) in plasma. The plasma SID directly alters blood pH and therefore independently changes acid–base status (17–20). The normal plasma SID of neonatal calves and adult cattle is approximately 40 mmol/L, and changes in plasma SID independently change plasma pH (20, 21). Two other factors, the plasma carbon dioxide tension (Pco_2_), and the total plasma concentration of non-volatile buffers (Atot), also independently change plasma pH (22). Interestingly, administration of an intravenous fluid changes plasma SID, and thereby changes plasma pH, by an amount that depends on the solution SID and volume relative to plasma volume. Intravenous fluid administration also dilutes the volume of distribution of plasma protein, the main non-volatile buffer in plasma, and this is accompanied by a decrease in plasma Atot and a direct decrease in plasma pH (23).

A physicochemical plot of plasma pH against the volume of solutions infused with differing SID provides an effective method for conveying the net alkalinizing or acidifying effect of intravenous crystalloid solution formulation on plasma pH [Figure 5; (19, 23)]. The approach was developed by Constable in 2004 (23), and a similar physicochemical approach was used in 2010 to plot Base Excess against the volume infused (24). The effect of infusing solutions of varying SID on plasma pH in calves can be calculated using Constable's 6-factor simplified strong ion equation (17, 18, 22) and applying experimentally determined values for Atot [0.343×(total protein in g/L)] and Ka (0.84 × 10^−7^) of calf jugular venous plasma and mean values from healthy calves for plasma pH (7.419), Pco_2_ (51 mm Hg), pK_1_' (6.105), *S* [0.0307 (mmol/L)/mmHg], total protein concentration (58 g/L), and SID (46.7 mmol/L) (25, 26). Figure 5 clearly indicates that 0.9% NaCl, 5% dextrose, or Ringer's solution decreases plasma pH in a dose dependent manner when infused intravenously, whereas intravenous administration of 1.3% sodium bicarbonate markedly increases plasma pH in a curvilinear manner. The overall effect of intravenous electrolyte solutions with an effective SID = 0 mmol/L, such as 0.9% NaCl or Ringer's solution, is a decrease in plasma pH because the strong ion acidosis causes a larger decrease in plasma pH than the independent effect of an increase in plasma pH due to concurrent non-volatile buffer ion alkalosis (21, 27). A surprising observation in calves is that lactated Ringer's solution, assuming L-lactate concentration is 14 or 28 mmol/L (28) and D-lactate is not metabolized, and acetated Ringer's solution, assuming gluconate is not metabolized, are predicted to produce a similar and very mild acidifying effect. This is because the intravenous administration of a crystalloid solution with an effective SID < 33 mmol/L will always be acidifying in healthy neonatal calves, whereas a crystalloid solution with an effective SID > 33 mmol/L will be alkalinizing (Figure 5), based on a plasma bicarbonate concentration of 33 mmol/L in healthy calves (26). Intravenous infusion of a solution with a SID of 40 mmol/L is mildly alkalinizing even though plasma SID remains constant at 40 mmol/L; this is because the addition of free water decreases plasma total protein and albumin concentrations, resulting in a mild increase in plasma pH. When a solution with a SID of 33 mmol/L is infused intravenously, the mild strong ion acidosis counterbalances the resultant mild non-volatile buffer ion alkalosis, with no net change in plasma pH. It should be noted that the decrease in plasma SID when 0.9% NaCl is administered depends upon the relative volume of the infused 0.9% NaCl solution to the initial extracellular space as well as the speed of 0.9% NaCl administration. Veterinarians should therefore be conversant with the SID of intravenous and oral fluid formulations as this plays an important role in optimizing fluid therapy.

The actual pH of commercially formulated intravenous fluids has no impact on its acidifying or alkalinizing potential; this statement does not hold in experimental conditions when strong acids or alkalis are infused, such as 300 mmol/L solutions of HCl or NaOH. The pH of commercially formulated solutions ranges from 5.5 to 8.0, and solution pH is determined primarily by the permeability of the polyvinyl chloride container to carbon dioxide (1). Diffusion of atmospheric CO_2_ through the container wall into the solution will decrease solution pH. Autoclaving of polyvinyl chloride bags as part of the sterilization process can produce enough acetate and formate to also lower solution pH (1).

### Isotonic, Hypertonic, and Hypotonic Intravenous Solutions

The tonicity of intravenous and oral electrolyte solutions is also an important clinical issue when treating critically ill neonatal calves and adult cattle, and an understanding of the difference between osmolarity and osmolality is required when selecting the optimal fluid therapy. **Osmolarity is calculated** from the mass concentration of particles in a solution assuming complete dissociation of ionic compounds such as NaCl and is expressed as mOsm/L of solution (in the case of 0.9% NaCl the solution is water). **Osmolality is measured** in the laboratory and expressed in units of mOsm/kg of solvent to accurately reflect the number of dissolved particles per kilogram of solvent. In plasma the solvent is plasma water that includes suspended proteins such as albumin and globulin. The measured plasma osmolality in neonatal calves and adult cattle is approximately 285 mOsm/kg [range, 275 to 295 mOsm/kg; (29, 30)]. In general terms, plasma osmolality is actively defended when plasma osmolality >295 or <275 mOsm/kg by increasing water intake or promoting free water excretion by the kidneys, respectively. The correct physiologic term when describing the tonicity of plasma is osmolality. This is because plasma osmolality accounts for the presence of plasma proteins and other substances that are suspended but not dissolved in plasma water, which is the solvent.

It should be noted that in clinical applications, intravenous fluid solution osmolarity is usually calculated from the stated concentrations. For example, 0.9% NaCl solution has a NaCl concentration of 154 mmol/L and a calculated osmolarity of 308 mOsm/L (2 × 154) assuming complete dissociation of NaCl. However, some of the sodium ions (cation) and chloride ions (anion) remain electrostatically attracted to each other in biological solutions, depending in part on the solution tonicity, so that the net result is that some of the NaCl is not completely dissociated in solution. Instead, a 0.9% NaCl solution contains separate sodium ions, chloride ions, and sodium-chloride particles (31). Experimental studies have determined that a 154 mmol/L solution of NaCl is about 86% dissociated, equivalent to an osmotic coefficient φ {phi} of 0.93 for NaCl (32), so that placing 154 mmol of NaCl in 1 L of water results in 132 mmol of sodium ions, 132 mmol of chloride ions, and 22 mmol of sodium-chloride particles = 286 mmol of particles (1.86 × 154) per L of water. In other words, the theoretical osmolarity of a 154 mmol/L solution of NaCl is 286 mOsm/L, which approximates a calculated osmolality of 286 mOsm/kg that is similar to the measured osmolality of plasma water in healthy cattle. A 0.9% NaCl solution distributes in plasma water when administered intravenously and is therefore isotonic when administered to cattle. Intravenously administered Ringer's solution (osmolarity, 309 mOsm/L), 1.3% NaHCO_3_ solution (osmolarity, 310 mOsm/L), and 5% dextrose solution (osmolarity, 278 mOsml/L) also distribute in plasma water and act as isotonic solutions when administered to cattle because they have calculated osmolalities of 287 mOsm/kg, 282 mOsm/kg, and 279 mOsm/kg, respectively. These osmolalities were calculated assuming that φ approximates 0.93 for Ringer's solution in plasma water, 0.91 for 1.3% sodium bicarbonate in plasma water (33), and 1.005 for dextrose in plasma water (34). It should be noted that a 5% dextrose solution can be obtained by adding 50 g of anhydrous dextrose with a molecular weight of 180 g to 1 L of water, or by adding 55 g of dextrose monohydrate, which is the most commonly used compound with a molecular weight of 198 g to 1 L of water.

On the above basis and in approximate terms, intravenous solutions administered to cattle can be defined as isotonic (275–295 mOsm/kg), hypotonic (<275 mOsm/kg), or hypertonic (>295 mOsm/kg) when osmolality is calculated from solution concentrations using the appropriate osmotic coefficient (9). It is not widely appreciated that some commonly used crystalloid solutions are hypotonic in calves and adult cattle when this classification system is used. For example, a buffered formulation of acetated Ringer's solution (Plasma-Lyte 148; calculated osmolarity, 295 mOsm/L; measured osmolality, 271 mOsm/kg) is mildly hypotonic, whereas lactated Ringer's solution (calculated osmolarity, 273 mOsm/L; measured osmolality, 256 mOsm/kg) is moderately hypotonic (1, 35, 36). Bovine erythrocytes are resistant to increases in plasma osmolality but susceptible to mild decreases in osmolality; intravenous fluids administered to cattle should ideally be isotonic or hypertonic because of the potential for hypotonic-induced hemolysis when large volumes of hypotonic solutions are rapidly administered (9). Nevertheless, the tonicity of lactated Ringer's solution is not low enough to raise clinical concerns of intravascular hemolysis in calves and adult cattle, although the additional free water is of theoretical concern when lactated Ringer's solution is rapidly administered in high volumes to endotoxemic animals.

In summary, lactated Ringer's solution and acetated Ringer's solution are polyionic crystalloid solutions that appear to be safe in neonatal calves and adult cattle. These two solutions have been widely used for treating moderate dehydration, electrolyte imbalances, and acidemia or alkalemia (9, 37). Unfortunately, lactated Ringer's solution and acetated Ringer's solution are not optimally formulated for ruminants and they are not usually adequate for treating neonatal calves or adult cattle with severe acidemia or alkalemia, hyponatremia, hypokalemia or hypochloremia.

### Distribution Space of Intravenously Administered Electrolytes

A critical factor in determining the amount of alkalinizing agent to provide to acidemic calves and adult cattle is an accurate knowledge of the total deficit. The total deficit of a plasma electrolyte in millimoles (mmol) is calculated as the deficit of the electrolyte in mmol per liter (Δmmol/L) multiplied by the distribution space for the electrolyte. The distribution space for sodium, chloride and bicarbonate is the extracellular fluid volume, which approximates 30% of BW in euhydrated adult cattle (38) and 44% in euhydrated neonatal calves (15). The total millimole deficit of sodium, chloride and bicarbonate for adult cattle or neonatal calves can be calculated as: deficit = (Δmmol/L) × (body weight in kg) × (0.30 or 0.44), respectively (7). Interestingly, the apparent bicarbonate space (apparent distribution volume for administered bicarbonate) in acidemic neonatal calves with diarrhea often appears to be greater than 0.44, and has been calculated to be 0.65 in 36 neonatal calves with naturally acquired diarrhea (39), 0.63 and 0.84 in 73 dehydrated diarrheic calves (40) and 0.73 and 0.78 in 11 healthy neonatal calves (41).

A notable clinical observation in neonatal calves, adult cattle, humans, and dogs is that the apparent bicarbonate space (ABS) is higher when the initial plasma bicarbonate concentration (*c*HCO_3_) is low. Based on a recent study in dehydrated calves with diarrhea (42), a rule of thumb would be to use an ABS value of 0.53 for dehydrated calves with diarrhea; however, when the results of jugular venous blood gas analysis are available, the apparent bicarbonate space (ABS) can be calculated from either the calculated value for venous *c*HCO_3_ or the measured value for Pco_2_, such that: ABS = 0.41 + 1.06/*c*HCO_3_ and ABS = 0.87 – 0.0082×Pco_2_ (42). Adult cattle are likely to require a similar adjustment of the ABS value for the initial plasma *c*HCO_3_.

## Intravenous Fluid Therapy in Neonatal Calves With Diarrhea

The major goals of intravenous fluid therapy in diarrheic calves are to: (a) expand the extracellular fluid volume and thereby restore venous return; (b) correct acidemia in calves with a jugular venous blood pH < 7.20 due to metabolic acidosis; (c) restore the suckle reflex and correct mental depression; (d) correct electrolyte abnormalities; (e) correct the energy deficit; and (f) facilitate repair of damaged intestinal epithelium (9, 10), although the latter goal is more appropriately addressed by oral administration of fresh cow's milk or an oral electrolyte solution (OES). Intravenous fluid administration should be monitored and intermittently reassessed (1, 9).

Studies based on the analyses of large datasets of diarrheic calves have shown that hyponatremia and unmeasured anions such as D-lactate are the most important contributors to acidemia in neonatal diarrheic calves (25, 43). Acidemia is also more common in dehydrated calves due to the hydration reduced decrease in glomerular filtration rate and therefore decreased ability to excrete a systemic acid load (44, 45). The preferred intravenous alkalinizing agent in neonatal calves with moderate acidemia, defined as blood pH < 7.20, is sodium bicarbonate. Metabolizable sodium salts of acetate and L-lactate result in a delayed increase in plasma bicarbonate, with L-lactate metabolism being slower than acetate metabolism (41). Gluconate and D-lactate are very slowly metabolized in neonatal calves and should not be administered as an alkalinizing agent (41, 46).

**Figure 6:**
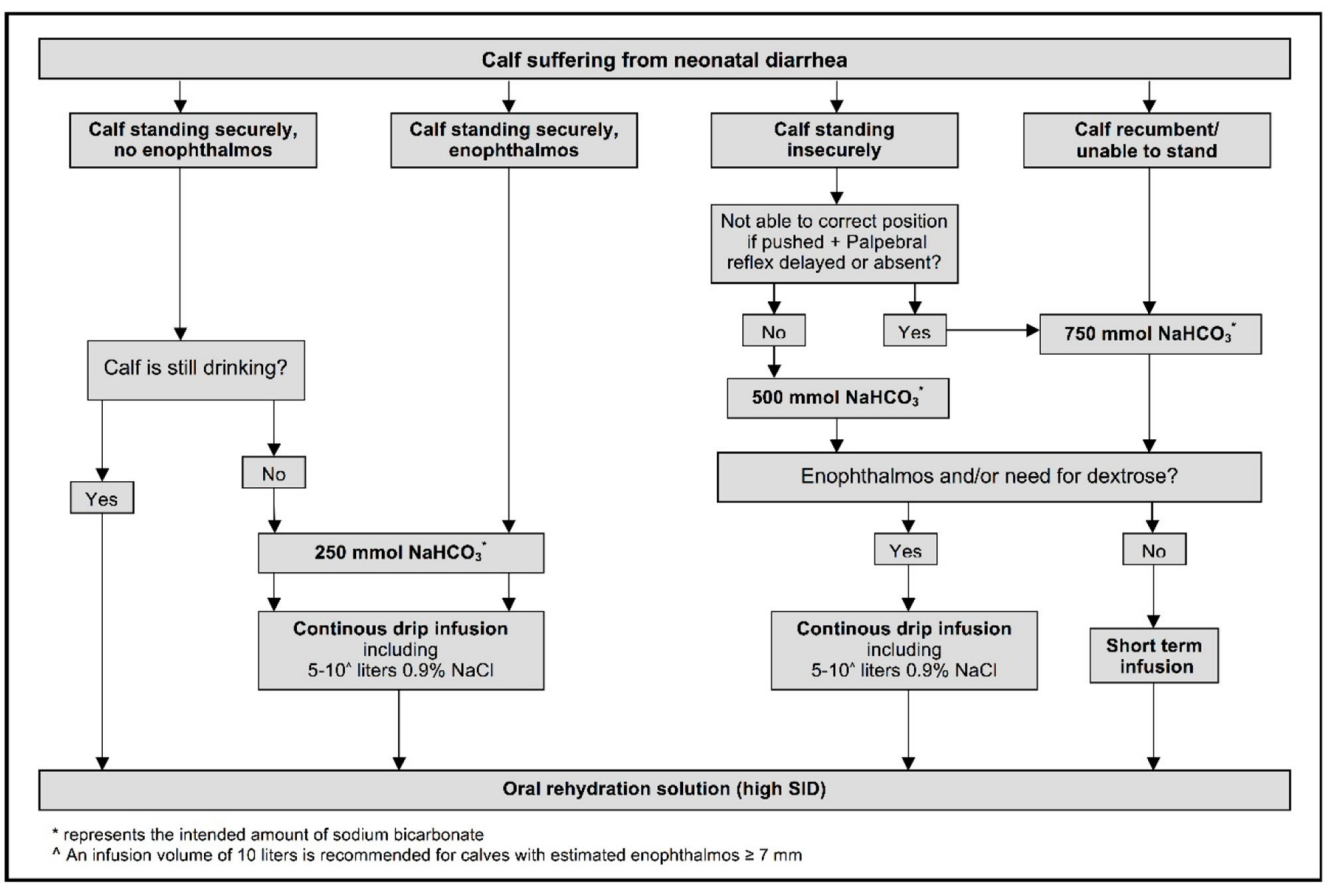
Decision tree for treating neonatal calves with diarrhea. The ability to stand is evaluated by lifting recumbent calves. Enophthalmos reflects a visible gap of 3–4 mm between the corneal surface of the eye and normal position of the lower eyelid. Reprinted with permission from: Trefz et al. (47).

An optimized decision tree has been developed for treating neonatal calves with dehydration and/or strong ion (metabolic) acidosis due to diarrhea [Figure 6; (47)]. The decision tree is based on ability to stand, hydration status (degree of enophthalmos), suckling strength, and presence or absence of a palpebral reflex. The need for oral fluids or intravenous administration of 250, 500, or 750 mmol of sodium bicarbonate, preferably as an isotonic solution with supplemental glucose added when indicated as part of the initial treatment, is identified by the decision tree (47). The decision tree also allows for short-term infusions of small-volume hypertonic NaHCO_3_ solutions with subsequent oral rehydration for treatment of calves with clinical signs of metabolic acidosis but no or minimal dehydration. The addition of 20 ml of 50% glucose solution to every liter of the initial intravenous fluid solution (providing 10 g glucose/L of solution) is beneficial on the first day of treatment of depressed diarrheic calves as hypoglycemia is common and associated with mortality (48, 49). However, subsequent glucose supplementation of intravenous solutions is not recommended as sustained intravenous glucose administration (10 g or 40 g glucose/L of solution) is associated with decreased voluntary milk intake (50).

### Isotonic and Hypertonic Sodium Bicarbonate Solutions

Isotonic sodium bicarbonate (1.3% NaHCO_3_ solution), an alkalinizing isotonic crystalloid solution, is the preferred treatment of neonatal calves with diarrhea, dehydration and moderate to severe acidemia. Isotonic sodium bicarbonate solution should be administered in calves with blood pH < 7.20 as a result of strong ion acidosis (51). Sodium bicarbonate exerts its alkalinizing action by buffering hydrogen ions, such that:

$$\begin{array}{l} {\text{HC}{\text{O}}_{3}}^{-}+{\text{H}}^{+}\text{ ⇌ C}{\text{O}}_{2}+{\text{H}}_{2}\text{O}, \end{array}$$

and increases plasma SID, as the effective SID of isotonic sodium bicarbonate solution is 155 mmol/L (23). Sodium bicarbonate is preferred to sodium L-lactate and sodium acetate solutions for treating neonatal calves with diarrhea and moderate to marked metabolic acidosis because it provides an immediate source of bicarbonate (39). Because diarrheic calves are usually hyponatremic, a mildly hypertonic 1.4% NaHCO_3_ solution (calculated osmolality, 304 mOsm/kg, Na concentration = 167 mmol/L) is preferred by some clinicians over a 1.3% NaHCO_3_ solution [calculated osmolality, 282 mOsm/kg; Na concentration = 155 mmol/L; 52].

Intravenous sodium bicarbonate solutions should not be administered to critically ill calves with respiratory disease on theoretical grounds because the additional CO_2_ generated may exacerbate hypercapnia and respiratory acidosis and decrease cerebrospinal fluid pH. A reasonable clinical recommendation is therefore to avoid administering intravenous hypertonic sodium bicarbonate solutions to calves with clinical signs of respiratory distress, such as marked thoracic excursions and abnormal auscultation or ultrasonographic findings. Attention should also be paid to early signs of hypercapnia such as muscle tremors and fasciculation while infusing hypertonic sodium bicarbonate. Nevertheless, studies in critically ill neonatal calves have not identified a clinically important effect of sodium bicarbonate infusion on increasing arterial Pco_2_ and further decreasing blood pH. The rapid administration of large volumes of sodium bicarbonate produces systemic alkalinization that may be accompanied by paradoxic cerebrospinal fluid (CSF) acidosis and adverse neurologic sequelae (52, 53). It should be noted that paradoxic CSF acidosis has only been reported in animals with controlled ventilation (7), and does not occur when sodium bicarbonate is administered to animals that control their own ventilation because any decrease in arterial blood pH due to an increase in arterial Pco_2_ results in a reflex increase in minute volume to combat the hypercapnia and respiratory acidosis (52).

Hypertonic sodium bicarbonate solutions are clinically effective for treating acidosis and hyperkalemia in neonatal calves with acute diarrhea, D-lactic acidosis, or mixed respiratory and strong ion (metabolic) acidosis (54–57). An 8.4% solution of sodium bicarbonate (2,000 mOsm/L) provides rapid alkalinization. An osmolarity of 2,000 mOsm/L is preferred because it provides 1 mmol of HCO_3_/ml of solution that permits simple calculation of the administered fluid volume. An 8.4% solution of sodium bicarbonate should not be administered faster than 1 ml/kg BW per minute when a total dose of bicarbonate (5 ml/kg BW) over 5 min in infused. This treatment regimen in normovolumic calves with experimentally induced mixed respiratory and metabolic acidosis caused a rapid increase in blood pH and improved cardiovascular status without inducing paradoxical cerebrospinal fluid acidosis (52). A subsequent study in calves with experimentally induced acidemia and hyperchloremic acidosis administered isotonic sodium bicarbonate solution over 4 h also failed to observe paradoxical cerebrospinal fluid acidosis (53). A 2008 study in dehydrated calves with naturally acquired diarrhea compared the resuscitative response of IV hypertonic sodium bicarbonate solution (8.4%, 10 ml/kg BW over 8 min) to IV hypertonic sodium chloride solution (5.9%, 5 ml/kg BW over 4 min); however, it should be noted that the hypertonic sodium chloride group received half the overall sodium load to that administered to the hypertonic sodium bicarbonate group. Both groups of calves also received 3 L of an OES 5 min after IV administration. The results of the study indicated that hypertonic sodium bicarbonate solution was more effective than hypertonic saline solution in correcting moderate acidemia and metabolic acidosis (56). Finally, a 2010 study of neonatal dehydrated calves with diarrhea compared the rapid IV administration of hypertonic (8.4%) and isotonic (1.3%) solutions of sodium bicarbonate administered at different volumes but similar sodium loads. The results of this study indicated that isotonic sodium bicarbonate solution was superior in rehydrating the calf because it contained more free water, whereas the rapid administration of the hypertonic solution was faster at correcting the acidemia and metabolic acidosis (54).

A major challenge with administering sodium bicarbonate solutions to dehydrated calves and adult cattle is the cost and availability of commercial products. This is because a high solution Pco_2_ tension must be maintained in solution to prevent dissociation of HCO_3_^−^ to CO_3_^2−^ and the precipitation of calcium carbonate within the solution. Carbon dioxide impermeable containers, such as glass, are needed to prevent carbon dioxide from leaving solutions of sodium bicarbonate. Commercially formulated bicarbonate solutions are therefore available only in glass containers that rarely exceed 1 L in volume. Bicarbonated Ringer's solution was formulated in Japan in 2005 in 500 mL plastic bottles and contained bicarbonate (25 mmol/L) and citrate (5 mmol/L) to prevent formation of calcium and magnesium precipitates (58), but this solution is now unavailable. Cattle veterinarians currently formulate sodium bicarbonate solutions for immediate use, often using 500 mL glass bottles of 5% sodium bicarbonate or 50–100 ml glass bottles of 8.4% sodium bicarbonate as a basis for solution formulation for administration to neonatal calves.

### Isotonic and Hypertonic Sodium Chloride Solutions

Isotonic sodium chloride (0.9% NaCl) is an acidifying isotonic crystalloid solution that has several shortcomings for treating neonatal calves with diarrhea and dehydration. Although isotonic and containing a sodium concentration of 154 mmol/L which is beneficial in calves with diarrhea as they are frequently hyponatremic (25), the effective SID of the solution is 0 mmol/L and consequently the solution is acidifying when administered intravenously (Figure 5). Moreover, 0.9% NaCl solution does not contain potassium, and neonatal calves with diarrhea have been thought for many years to be potassium deficient (59), despite some calves having hyperkalemia (9, 45, 60, 61). The assumption that diarrheic calves have whole body depletion of potassium has been recently challenged in a study of calves with acute diarrhea and marked dehydration and acidemia, in that diarrheic calves had similar skeletal muscle potassium concentrations to healthy calves (62). Possibly whole-body potassium deficiency only occurs in calves with chronic diarrhea, or does not occur at all.

Small volumes (4–5 ml/kg BW) of hypertonic NaCl solutions (7.0 to 7.5% NaCl) have been extensively investigated for treating hypovolemic shock in domestic animals and humans. The results of physiologically focused studies indicate that hypertonic saline increases plasma volume by osmotically driving free water to move from the intracellular space to the extracellular space where the translocated free water is retained by the additional sodium load. This increases the cardiac output despite a transient decrease in cardiac contractility, increases mean circulatory filling pressure, systemic oxygen delivery, and mean arterial blood pressure, while decreasing total peripheral vascular resistance and pulmonary vascular resistance (63, 64). The resultant increase in glomerular filtration rate restores urine output and acid–base balance returns toward normal due to improved organ perfusion and renal clearance of protons (65).

The most commonly used hypertonic saline formulation for the rapid resuscitation of animals with hypovolemia is 7.2% NaCl (2,460 mOsm/L). Hypertonic saline should be intravenously administered at 4–5 ml/kg BW over 4–5 min, equivalent to 1 ml/kg BW per minute. Faster administration rates directly cause vasodilation and decreased cardiac contractility, resulting in hemodynamic collapse (63, 64). Similar to the intravenous administration of high-volume 0.9% NaCl solution that has an effective SID = 0 mmol/L, small-volume 7.2% NaCl solution induces a mild strong ion acidosis and transient decrease in pH of up to 0.08 pH units that rapidly dissipates with time and is thought to not be clinically important (65).

**Figure 7:**
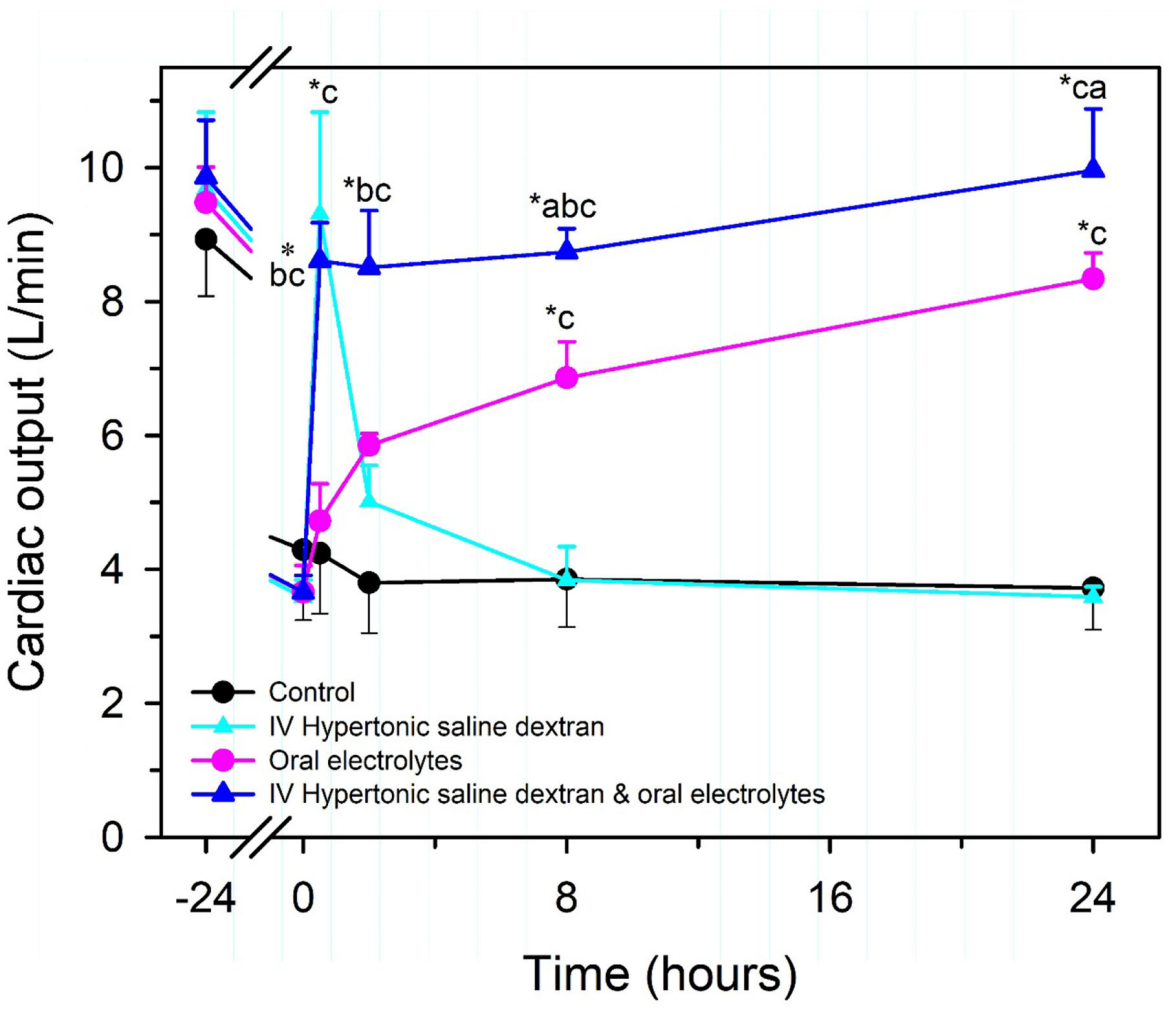
Cardiac output in healthy neonatal calves (time = −24 h) and after experimentally induced diarrhea and dehydration (time = 0 h). Calves were then randomly assigned to no treatment (Control; black circles, *n* = 4) or treatment with an intravenous hypertonic saline-dextran (HSD) solution (2,400 mOsm/L NaCl in 6% dextran-70 at 4 ml/kg BW once over 4 min; cyan triangles, *n* = 4), an isotonic alkalinizing oral electrolyte solution (55 ml/kg BW every 8 h for three treatments; pink circles, *n* = 4); or a combination of intravenous HSD and oral treatments (blue triangles, *n* = 4). *P < 0.05, compared with *t* = 0 value; ^a^*P* < 0.05 compared with hypertonic saline dextran group; ^b^*P* < 0.05 compared with oral electrolyte solution; and ^c^*P* < 0.05, compared with the untreated control group. Adapted, with permission, from: Constable et al. (66).

**Figure 8:**
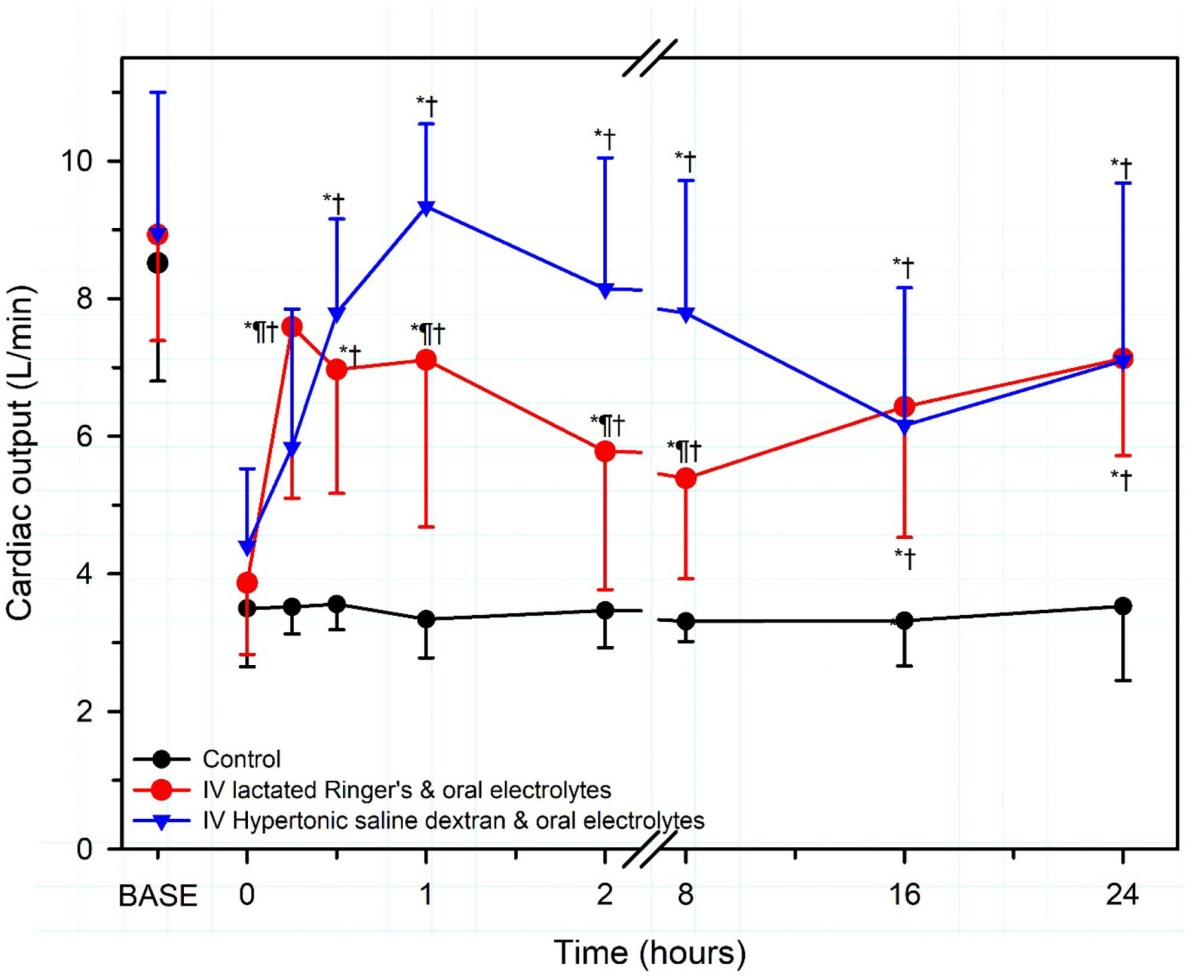
Cardiac output in severely dehydrated diarrheic calves treated with no fluid (Control, black circles; *n* = 5); intravenous lactated Ringer's solution (80 ml/kg BW for 1 h then 4 ml/kg BW for 7 h) and an oral electrolyte solution (OES; 40 ml/kg BW at 8 and 16 h; red circles, *n* = 5); or intravenous hypertonic saline-dextran solution (HSD, 2,400 mOsm/L NaCl in 6% dextran-70 at 4 ml/kg BW once over 4 min) combined with an OES (60 ml/kg BW every 8 h for three treatments, blue triangles, *n* = 5). Base refers to baseline, the time before induction of diarrhea and dehydration. *significantly different from time = 0 h; ^†^significantly different from control group at the same time; ¶significantly different from the lactated Ringer's group at the same time. Revised, with permission, from: Walker et al. (13).

Intravenous hypertonic saline (7.2–7.5% NaCl) administered through an 18 g needle at 4–5 ml/kg BW over 4–5 min, alone or accompanied by the colloid dextran-70, has been used to successfully resuscitate dehydrated calves with diarrhea [Figure 7; (13, 51, 57, 66–68)]. Dextran accompanied hypertonic saline in the initial studies in calves because it produces sustained plasma volume expansion in other shock models (13, 66, 67); however, subsequent studies have indicated that a beneficial response can be obtained without dextran (8, 51, 68, 69). The clinical response to treatment is optimized if calves receive 2–3 L of an isotonic OES by esophageal intubation immediately before hypertonic saline is administered (7, 65). The fastest rate of resuscitation is produced by combined administration of intravenous hypertonic saline and an OES, based on the speed of increase in plasma volume, stroke volume, and cardiac output (13, 51, 66), and the rate of resuscitation with the combined treatment is faster than that provided by lactated Ringers solution administered at 80 mL/kg during the first hour of infusion [(13, 51); Figure 8]. Consequently, when treating dehydrated diarrheic calves that are not markedly acidemic (i.e., blood pH anticipated to be >7.20), the rapid infusion of small volumes of hypertonic saline is preferred over the infusion of large volumes of lactated Ringer's solution. Moreover, hypertonic saline administration results in greater retention of the administered sodium and sustained resuscitation, whereas lactated Ringers solution or 0.9% NaCl solution increase urinary sodium and free water loss (13, 66, 68).

### Lactated Ringer's Solution

Lactated Ringer's solution is a moderately hypotonic (256 mOsm/kg) balanced, polyionic, crystalloid solution containing physiological concentrations of K^+^, Ca^2+^, and Cl^−^, and concentrations of Na^+^, L-, and D-lactate (CH_3_CH(OH)COO^−^) that differ from that of cattle plasma. The solution is commonly administered to calves with mild to moderate dehydration that can stand but have a poor or no suckle response (8, 9, 13, 37). Lactated Ringer's solution is widely regarded as mildly alkalinizing because the strong anion L-lactate can be removed from plasma via gluconeogenesis in the cytosol to produce glucose, an uncharged molecule, while consuming two protons (41), such that:

$$\begin{array}{l} 2\text{C}{\text{H}}_{3}\text{CH}\left(\text{OH}\right)\text{CO}{\text{O}}^{-}+2{\text{H}}^{+}↔{\text{C}}_{6}{\text{H}}_{12}{\text{O}}_{6} \end{array}$$

A variable amount of L-lactate is converted to pyruvate by lactate dehydrogenase in the cytosol, thereby consuming a proton, and then oxidized in mitochondria through the citric acid pathway to produce carbon dioxide and water (41); the final metabolic products are not strong anions and consequently the plasma SID and blood pH increase. The overall metabolic fate of L-lactate via oxidation can be summarized as:

$$\begin{array}{l} 2\text{C}{\text{H}}_{3}\text{CH}\left(\text{OH}\right)\text{CO}{\text{O}}^{-}+2{\text{H}}^{+}+6{\text{O}}_{2}\to 6\text{C}{\text{O}}_{2}+\;6{\text{H}}_{2}\text{O}. \end{array}$$

Finally, a very small amount of L-lactate in plasma is excreted in the urine, particularly when plasma L-lactate concentrations are high, and this also represents a loss of a plasma strong anion and subsequent increase in plasma SID.

Lactated Ringer's solution is typically a racemic mixture of L-lactate and D-lactate that historically contained approximately equivalent concentrations of L-lactate and D-lactate isomers. The L-lactate isomer predominates in most currently available commercial products (28). L-lactate is rapidly metabolized to bicarbonate in mammals, including neonatal calves; however, mammals have minimal D-lactate dehydrogenase. This results in slowed clearance of D-lactate that occurs primarily by excretion through the urinary system (41). Racemic DL-lactate solutions therefore have an effective SID less than the calculated value of 28 mmol/L for lactated Ringer's solution. The net result is that lactated Ringer's solution has an effective SID of 14–28 mmol/L and is neutral to slightly acidifying from a physicochemical perspective when administered to healthy calves with normal acid–base balance [Figure 5; (70, 71)] because the mean plasma bicarbonate concentration of healthy calves is 33 mmol/L (26). However, it should be recognized that if the lactated Ringer's formulation contains only the L-isomer, lactated Ringer's solution will be alkalinizing whenever the plasma bicarbonate concentration is <33 mmol/L, which is frequently the case in diarrheic calves (25, 43, 49). Severely dehydrated calves can have increased blood L-lactate concentrations and a 50% decrease in the rate of L-lactate metabolism (72). The latter finding suggests that it may be clinically irrational to add additional L-lactate to hyperlactatemic neonatal calves.

Increasing the L-lactate concentration of lactated Ringer's solution should produce a mild to moderate alkalinizing effect, and in healthy calves the addition of L-lactate at 56 or 84 mmol/L to a 0.9% NaCl solution provided a similar alkalinizing effect to that provided by adding bicarbonate at 56 or 84 mmol/L (71). It is likely that the clinical effectiveness of lactated Ringer's solution in neonatal calves can be improved by increasing the L-lactate concentration, eliminating the D-lactate isomer, and increasing the Na concentration to 140 mmol/L.

### Acetated Ringer's Solution

Acetated Ringer's solution is a slightly hypotonic (271 mOsm/kg) balanced, polyionic crystalloid solution containing physiological concentrations of Na^+^, K^+^, Mg^2+^, and Cl^−^ and concentrations of acetate (CH_3_COO^−^) and gluconate (CH_2_(OH){CH(OH)}_4_COO^−^) that differ markedly from that of cattle plasma. Acetated Ringer's solution is widely regarded as a mildly alkalinizing solution because acetate is metabolized to carbon dioxide and water while consuming a proton (41), such that:

$$\begin{array}{l} \text{C}{\text{H}}_{3}\text{CO}{\text{O}}^{-}+{\text{H}}^{+}+{\text{O}}_{2}\to 2\text{C}{\text{O}}_{2}+\;2{\text{H}}_{2}\text{O}. \end{array}$$

Acetated Ringer's solution is alkalinizing because acetate, a metabolizable strong anion, increases plasma SID when metabolized. It is not widely appreciated that the gluconate in acetated Ringer's is problematic because calves (and presumably all mammals) do not metabolize, or very slowly metabolize, gluconate (41, 46); consequently, gluconate acts as a strong anion when administered intravenously, counteracting any potential alkalinizing effects of acetate (46). The net result is that acetated Ringer's solution is neutral to slightly acidifying from a physicochemical perspective (Figure 5) when administered at 20 ml/kg BW per hour to diarrheic calves (73). Contrary to popular belief, acetated Ringers solution is theoretically slightly acidifying in calves because its effective SID of 28 mmol/L is less than the mean plasma bicarbonate concentration for calves [33 mmol/L; (26)]. However, it should be recognized that acetated Ringer's solution will be alkalinizing whenever the plasma bicarbonate concentration is much lower than 33 mmol/L, which is frequently the case in diarrheic calves (25, 43, 49), because metabolism of acetate consumes a proton which is equivalent to generating a bicarbonate molecule.

Acetated Ringer's solution is commercially available in three formulations in North America, Plasma-Lyte A, Plasma-Lyte 148, and Normosol-R. All three have similar formulations except Plasma-Lyte A and Normosol-R have a solution pH of 7.4, compared to Plasma-Lyte 148 that has a solution pH of 6.0 (1); however, the solution pH difference is not clinically significant. Acetated Ringer's solution has the clinical advantage over lactated Ringer's solution in that its sodium concentration (140 mmol/L) is approximately the same as that of neonatal calves. The sodium concentration in lactated Ringer's solution (130 mmol/L) is appreciably lower. An additional advantage of Acetated Ringer's solution is that the rate of acetate metabolism to bicarbonate is faster than that of L-lactate (39, 41, 70). The main disadvantage of acetated Ringer's solution is that the solution is vasodilatory due to the inclusion of acetate (74–76), resulting in mild decreases in mean arterial blood pressure during infusion. This reduction may be clinically important in critically ill neonatal calves. It is likely that the clinical effectiveness of acetated Ringer's solution in neonatal calves can be improved by eliminating gluconate from the formulation. Studies that compare the ability of equal sodium and strong anion loads of acetated Ringer's solution (without gluconate) and lactated Ringer's solution (without the D-lactate isomer) to alkalinize and expand the plasma volume in neonatal calves with diarrhea are indicated.

### Other Intravenous Solutions

Many intravenous solutions for fluid therapy of calves with diarrhea have been proposed and a consensus recommendation for home-formulated intravenous solutions are that they should contain approximately 150 mmol/L of sodium, 5 mmol/L of potassium and at least 50 mmol/L of bicarbonate or bicarbonate precursors such as L-lactate or acetate (9). Other intravenous solutions (Darrow's solution, McSherry's balanced electrolyte solution, Tromethamine, and Carbicarb) have been administered to neonatal calves with diarrhea. Some of these solutions have theoretical advantages over existing solutions, but these solutions are not currently used because commercially available formulations are not available.

Darrow's solution is an isotonic polyionic solution that was formulated in 1946 for administration to dehydrated human infants (77). Darrow's solution has been administered to calves but when compared to acetated Ringer's solution, Darrow's solution has a low sodium, high potassium and high lactate concentrations and does not contain magnesium. McSherry's balanced electrolyte solution is an isotonic polyionic solution that was formulated in 1954 for parenteral administration to dehydrated calves with diarrhea (78). This appears to be an excellent parenteral fluid that deserves research investigation (7).

Tromethamine is an organic amine (THAM, *t*ris-*h*ydroxymethyl *a*mino*m*ethane, (CH_2_OH)_3_C-NH_2_) formulated as an isotonic solution (300 mmol/L) that is a safe and effective buffer in biological fluids. After intravenous administration to mammals, approximately 70% of the uncharged tromethamine compound is immediately protonated in plasma at physiologic pH to the strong cation (CH_2_OH)_3_C-NH_3_^+^ (79), with the net buffer equation being:

$$\begin{array}{l} {\left(\text{C}{\text{H}}_{2}\text{OH}\right)}_{3}\text{C}\text{-N}{\text{H}}_{2}+{\text{H}}^{+}\text{ ⇌ }{\left(\text{C}{\text{H}}_{2}\text{OH}\right)}_{3}\text{C}\text{-}{\text{N}{\text{H}}_{3}}^{+}. \end{array}$$

The unprotonated form of tromethamine can cross cell membranes and theoretically buffer the intracellular compartment. Tromethamime should be administered in conjunction with electrolytes as its administration without additional electrolytes leads to hyponatremia (79). Tromethamine does not currently offer any documented advantages to the administration of an isotonic sodium bicarbonate solution in acidemic neonatal calves, and consequently its administration is not recommended.

Carbicarb® is an isotonic buffer (300 mOsm/L) that contains equimolar amounts of disodium carbonate (Na_2_CO_3_) and sodium bicarbonate (NaHCO_3_) that completely dissociate in plasma; however, carbonate generates HCO_3_ when buffering acidemic blood, and the generated HCO_3_ can also buffer the proton, such that:

$$\begin{array}{l} {\text{HC}{\text{O}}_{3}}^{-}+{\text{C}{\text{O}}_{3}}^{2-}+3{\text{H}}^{+}+3\text{N}{\text{a}}^{+}\text{ ⇌ }2\text{C}{\text{O}}_{2}+2{\text{H}}_{2}\text{O}+\;3\text{N}{\text{a}}^{+} \end{array}$$

Carbicarb® was anticipated to decrease the magnitude and occurrence of hypercapnia when administered to animals with mixed metabolic and respiratory acidosis (80, 81). Potential clinical advantages of Carbicarb® are only apparent in animals with limited ventilatory ability or controlled ventilation (7). Carbicarb® has been administered intravenously to neonatal calves with diarrhea (80) and newborn calves with metabolic acidosis when rapid alkalinization of neonatal calves with diarrhea is required (81). In these studies, Carbicarb® did not provide a clinically relevant advantage over isotonic sodium bicarbonate administration and its use is therefore not currently recommended.

## Intravenous Fluid Therapy in Calves With Clinical Coccidiosis

Most fluid therapy studies have been conducted on neonatal calves that are < 21 days of age because of the high incidence of diarrhea during the first three weeks of life. Twenty-one days of age is also an appropriate physiologic cut-point for categorization, as calves that are conventionally raised (non-veal) are regarded physiologically as a monogastric from birth to 21 days of age, in a transition period from 3 to 8 weeks of age, and as a ruminant beyond 8 weeks of age. These age categories are associated with different forestomach flora and plasma concentrations of glucose, B-OH butyrate, and short chain volatile fatty acids, consistent with the change from the primary source of energy transitioning from milk to forage.

Coccidiosis, and to a lesser extent giardiasis, are common causes of large intestinal diarrhea in calves from 28 days to 6 months of age. Only a few peer-reviewed studies have been published that describe the clinicopathological changes in calves with clinical coccidiosis (82, 83). Clinical experience indicates that calves with clinical coccidiosis are rarely acutely dehydrated because they increase their water intake in response to increased fecal water loss; however, hyponatremia can be much more severe in calves with coccidiosis than in neonatal calves with diarrhea due to cryptosporidia, rotavirus, coronavirus, and enterotoxigenic *E. coli*. Another important difference in bovine coccidiosis is that hyponatremia is usually accompanied by hypochloremia; this is because similar percent reductions in plasma sodium and chloride concentrations are common in infectious diseases that primarily affect the large intestine (84). Plasma sodium concentrations of 125 mmol/L are common in calves with clinical coccidiosis, occasionally decrease to 115 mmol/L, and in extreme cases may be 100–105 mmol/L and even below, with similar reductions in plasma chloride concentrations, so that plasma SID remains similar to reference values or is mildly decreased. Another important clinicopathologic difference in bovine coccidiosis to neonatal calf diarrhea is that blood pH and plasma bicarbonate concentrations are usually minimally decreased (83).

There do not appear to be any standardized clinical studies on the response to intravenous fluid administration in calves with coccidiosis. The reported clinicopathological changes in clinical coccidiosis suggest that a NaCl solution would provide the preferred intravenous fluid for calves with plasma sodium concentration < 125 mmol/L; however, the potential for calves with coccidiosis to have severe chronic hyponatremia (i.e., plasma sodium concentrations below 115 mmol/L for at least 24 h) raises concern over the rapid return of plasma sodium concentrations to reference values, which has been associated with pontine demyelinization in other species, and possibly manifest as “nervous coccidiosis” in treated calves. A practical “two buckets” approach to fluid therapy in calves with coccidiosis has therefore been developed. The plasma sodium concentration is measured in the morning and two buckets are placed in the stall for calves with plasma sodium concentration > 125 mmol/L; one that has plain water, and a second bucket that contains 150 g of NaCl per 10 L (sodium concentration, 256 mmol/L). Calves with clinical coccidiosis usually prefer the water bucket containing NaCl and their plasma sodium concentration gradually returns to the reference range. Calves with a plasma sodium concentration < 125 mmol/L are best treated intravenously over the following 24 h with a NaCl solution that has a sodium concentration 10–15 mmol/L higher than the measured plasma concentration. The calf stall should contain only one bucket of water with no added NaCl. This process is repeated each morning until the plasma sodium concentration is > 125 mmol/L, at which time intravenous fluid therapy is discontinued and the “two buckets” approach is implemented. Oral electrolyte solutions formulated for calves with neonatal diarrhea are unsuitable for oral rehydration of calves with clinical coccidiosis as these solutions generally contain insufficient amounts of sodium to correct hyponatremia and furthermore contain considerable amounts of carbohydrates and alkalinizing agents, neither of which are required or indicated in these patients.

## Intravenous Fluid Therapy in Neonatal Calves With Sepsis

**Figure 9:**
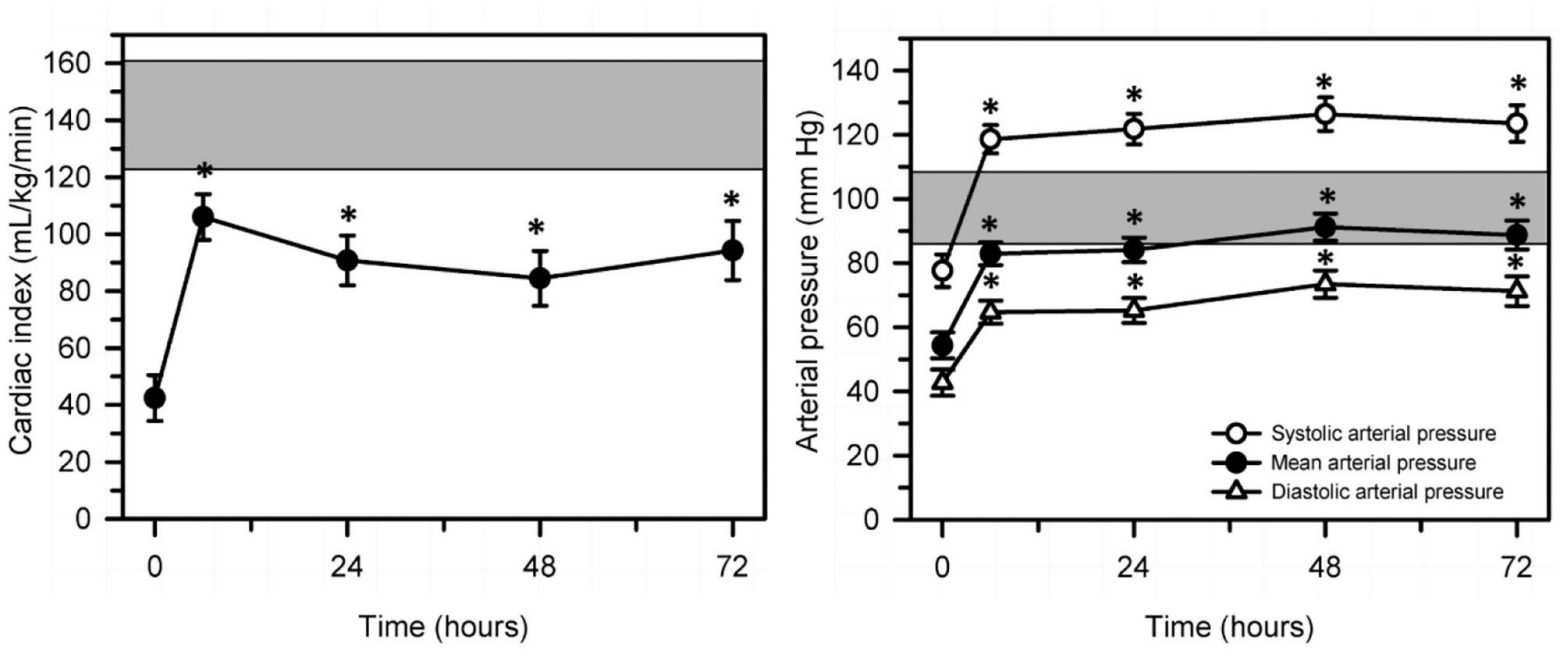
Changes in echocardiographically determined cardiac index (left panel) and systolic, mean, and diastolic arterial pressure (right panel) over 72 h after the start of a standardized treatment protocol in 20 septic calves that included intravenous fluids and vasopressors. Data are presented as least squares mean and SE. The number of calves alive at each measurement time were 20 at 0 and 6 h, 17 at 24 h, 14 at 48 h, and 12 at 72 h. The gray shaded rectangle is the 95% confidence interval for the mean value for 10 healthy calves of similar age and body weight (right panel, mean value for mean arterial pressure). *P < 0.0125 compared to time = 0 h value. Reproduced with permission from: Naseri et al. (86).

**Figure 10:**
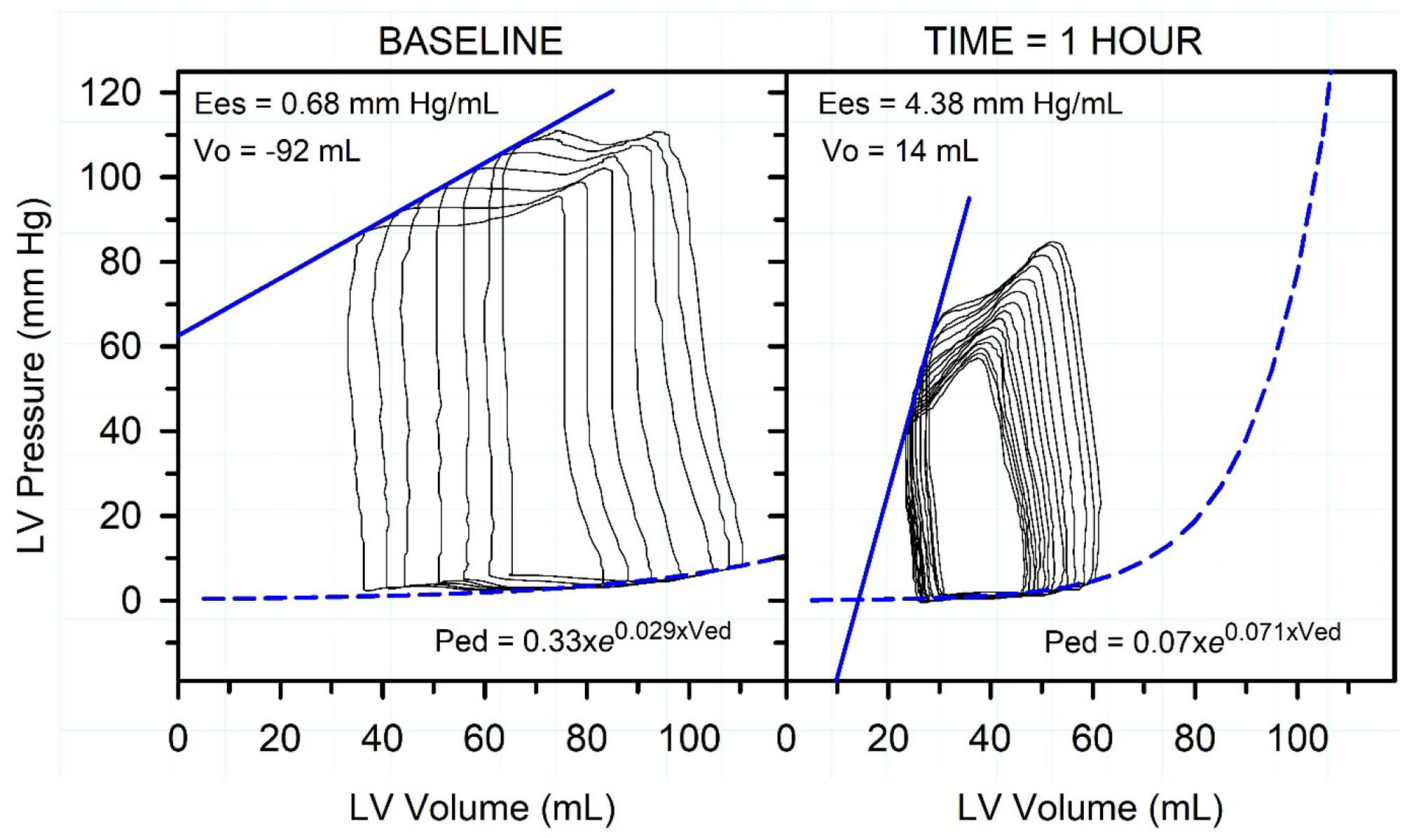
Representative pressure-volume loops, end-systolic pressure-volume relationship (straight blue line), and end-diastolic pressure-volume relationship (dashed exponential blue line) in an endotoxemic calf. Caudal vena-caval occlusion was used to decrease preload while left ventricular (LV) pressure and volume were recorded. The left panel is before endotoxin was administered. The right panel is 1 h after start of endotoxin administration. Note that the administration of endotoxin markedly decreased end-systolic pressure and increased End-systolic elastance (Ees; the gold standard index of cardiac contractility), decreased stroke volume and end-diastolic volume, slightly increased LV diastolic stiffness, and increased heart rate (not shown). The figure indicates that systemic arterial hypotension is the dominant cardiovascular derangement in acute endotoxemia. Reproduced with permission from: Constable (89).

Sepsis and septic shock have been documented to be associated with clinically relevant cardiovascular changes in humans and domestic animals. In children, septic shock is now defined as “*severe infection leading to cardiovascular dysfunction, including hypotension, need for treatment with a vasoactive medication, or impaired perfusion*” (85). This is differentiated clinically from sepsis-associated organ dysfunction that represents “*severe infection leading to cardiovascular and/or non-cardiovascular organ dysfunction*” (85). The cardiovascular changes include a relative or absolute decrease in central blood volume, systolic and diastolic alterations of left ventricular and right ventricular function, marked peripheral vasodilation accompanied by systemic hypotension, and increased vascular permeability (86). Studies in neonatal calves with naturally acquired sepsis [(86); Figure 9] or experimentally induced endotoxemia [(87–89); Figure 10] indicate that impaired cellular metabolism and circulatory dysfunction, not systolic dysfunction, are the primary clinical abnormalities in septic neonatal calves. Impaired cellular metabolism is characterized by sustained hyperlactatemia, azotemia, hypoglycemia and hypothermia and is most likely due to inadequate nutrient delivery at the cellular level (86). Hyperlactatemia is a biomarker of tissue hypoxia and abnormal cellular metabolism, as well as disease severity and mortality in critically ill humans (90) and domestic animals, including calves and adult cattle (49, 91–93). Circulatory dysfunction in septic calves is characterized by decreased preload, manifest as low left ventricular end diastolic volume due to maldistribution of venous blood (relative hypovolemia), and decreased afterload, manifest as low mean arterial blood pressure (86). Current treatment protocols for septic calves are frequently successful, whereas the treatment success of calves in septic shock is markedly lower and best characterized as poor. Early diagnosis of septic shock in calves is likely to have the greatest impact on improving treatment outcomes.

Veterinarians administering intravenous fluids to calves in septic shock need to adjust standard monitoring and treatment protocols because of the presence of systemic hypotension and maldistribution of venous blood. Recent studies in septic human patients have identified concerns with bolus fluid resuscitation in the first hour (20–40 ml/kg) using isotonic polyionic crystalloid solutions (94–96). This fluid administration protocol is associated with increased mortality due to development of cerebral and pulmonary edema (96), most likely due to increased luminal pressures in capillary beds and increased vascular permeability. Traditional high volume isotonic polyionic crystalloid fluid resuscitation of septic neonatal calves should therefore utilize slower rates of administration of up to 20 ml/kg BW per hour. Vasopressor therapy (norepinephrine at 10 μg/kg BW/min for 60 min) should be considered in neonatal calves with mean arterial blood pressure < 65 mm Hg after initial fluid therapy (86). More work is needed in this area as the 2020 guidelines for treating hypotensive shock in children in low resource settings recommends administering isotonic crystalloid solutions in the first hour at up to 40 ml/kg, and vasopressor agents (epinephrine or norepinephrine, and possibly vasopressin) for systemic hypotension (85). Fresh whole blood, if available, has theoretical advantages in the initial treatment of septic calves, including improved oxygen delivery (hemoglobin) and buffering (hemoglobin, plasma proteins) as well as increased plasma immunoglobulin concentrations, but contemporary randomized clinical trials using a goal directed resuscitation approach have not been performed in calves.

An alternative strategy would be to administer low volume hypertonic crystalloid fluids for the initial resuscitation of septic calves, particularly those in septic shock. Hypertonic saline solutions (7.2% NaCl, 2,400 mOsm/L, 4 ml/kg BW) have been safely administered to endotoxemic calves, and the rapid intravenous administration of hypertonic saline over 4 min successfully, but transiently, resuscitated calves with experimentally induced endotoxic shock (87, 88). Nevertheless, rapid infusion of large-volume isotonic saline was clinically superior to small-volume hypertonic saline-dextran in calves with experimentally induced acutely endotoxemia (87). A 2014 study in endotoxemic swine evaluated the effect of hypertonic solutions of sodium lactate (11.2%) and sodium bicarbonate (8.4%) at 5 ml/kg per hour administered over 270 min (97). The hypertonic sodium lactate solution increased cardiac output and mean arterial blood pressure while improving oxygenation, relative to hypertonic sodium bicarbonate or isotonic saline (0.9% NaCl). In contrast, a 2016 study in sheep with septic shock, the administration of a hypertonic lactate solution (500 mmol/L) resulted in a shorter survival time than administration of hypertonic saline solution [3.0%; (98)]. The latter finding raises concerns about the use of hypertonic sodium lactate solutions in calves with septic shock. Additional studies appear indicated comparing hypertonic sodium chloride, sodium bicarbonate, and sodium lactate solutions as part of the initial resuscitation of septic calves. Until such studies are completed, isotonic polyionic fluids should be administered intravenously at < 20 mL/kg BW per hour to septic calves, and consideration should be given to administering hypertonic saline (7.2%, 4–5 ml/kg BW over 5 min) intravenously for the initial resuscitation of calves in septic shock.

The supplementation of intravenous fluids with glucose should always be considered in septic calves. Hypoglycemia occurs commonly in septicemic calves with diarrhea due to increased glucose utilization and decreased appetite, and its occurrence is associated with increased mortality (48, 49, 88). The addition of 20 ml of 50% glucose solution to the first liter of the initial intravenous fluid solution (providing 10 g glucose/L of solution and a solution glucose concentration of 56 mmol/L) should be routinely implemented in the initial treatment of septic calves as hypoglycemia is common and associated with mortality in critically ill calves (49). Due to the difficulties in predicting the outcome of intravenous dextrose administration in septic calves, it is advisable to regularly check the blood glucose concentration with a point of care glucometer validated for use in cattle (99). This will permit adjustment of the rate of intravenous dextrose infusion with the objective being to restore and maintain euglycemia.

## Intravenous Fluid Therapy in Adult Cattle

Critically ill adult ruminants differ markedly in their acid-base and electrolyte response to those discussed above in critically ill neonatal calves with diarrhea. Cattle with abdominal diseases that require surgical correction, such as left displaced abomasum, right displaced abomasum, abomasal volvulus, abomasal reflux, small intestinal obstruction, and gastrointestinal hypomotility/atony, are usually dehydrated with the major clinicopathologic changes being a hypochloremic, hypokalemic, metabolic alkalosis due to sequestration of chloride and potassium in the proximal portion of the gastrointestinal tract (38, 100). Moreover, sick lactating dairy cattle, particularly in early lactation, almost always have subclinical hypocalcemia because of the reduction in feed and calcium intake relative to the loss of calcium in milk. As a result of these clinicopathological changes, the preferred intravenous solution in adult cattle should be acidifying and polyionic. An exception to this recommendation is cattle with ruminal acidosis due to excessive consumption of rapidly fermentable starches such as cereal grains that results in metabolic acidosis. Acidemia due to metabolic acidosis can occur in other conditions such as esophageal obstruction (choke), listeriosis with cranial nerve paralysis resulting in excessive loss of saliva by drooling, advanced cases of abomasal volvulus and small intestinal obstructive conditions such as intussception, volvulus or obstruction, and cattle with severe diarrhea or renal failure.

Laboratory evaluation of critically ill cattle is helpful as the magnitude of clinicopathologic abnormalities is difficult to predict based on clinical signs, with the exception being clinical hypocalcemia and clinical hypomagnesemia. However, laboratory evaluation is often unavailable in a timely or low-cost manner, and under these circumstances, the administration of Ringer's solution, 0.9% NaCl or 7.2% NaCl solutions is recommended for correction of the assumed strong ion (metabolic) alkalosis. Subclinical hypocalcemia is rare in sick beef cattle because many examined animals are not lactating, and if they are lactating the milk volume suckled by the beef calf is much lower than the average daily milk production of today's dairy cow.

### Ringer's Solution

Ringer's solution should be regarded as the default intravenous fluid for dehydrated adult ruminants, particularly lactating dairy cows, because this intravenous fluid formulation provides the best available solution to treat hypochloremic, hypokalemic, metabolic alkalosis, along with the oral administration of potassium in animals that are inappetent (38, 100). Ringer's solution is an isotonic, balanced, polyionic, acidifying crystalloid solution that contains concentrations of Na (147 mmol/L) K (4 mmol/L), Ca (2 mmol/L), and Cl (155 mmol/L). Ringer's solution is acidifying because its effective SID = 0 mmol/L (Figure 5). Intravenous administration of a fluid with a SID of 0 mmol/L will decrease plasma SID (normal SID ≈ 40 mmol/L) and therefore directly decrease plasma pH. At reference values for adult cattle, a 1 mmol/L decrease in SID directly cases a decrease in plasma pH of approximately 0.016 (20, 25).

Intravenous administration of equivolume Ringer's solution and 5% dextrose solutions (16) or equivolume lactated Ringer's solution and 4.4% dextrose solutions (101) have been recommended for rehydrating dehydrated adult cattle. Unfortunately, the former formulation produced a marked hyperglycemia during infusion (plasma glucose concentration peaked at 350 mg/dl) (16) that would be expected to decrease food intake (30) and promote renal diuresis as the plasma glucose concentration exceeds the renal threshold for glucose excretion [(102); **Figure 13**]. Care therefore needs to be taken when adding dextrose to Ringer's solution.

The biggest challenges with administering Ringer's solution to dehydrated adult cattle are the cost and availability of commercial products. Ringer's solution is not available for purchase in North America. In countries where commercial products are available (such as Europe and New Zealand), the price of Ringer's solution per Liter is very high relative to 0.9% NaCl solutions, and the largest volume available is 3 L. Ringer's solution can be formulated at relatively low cost by taking a 5 L bag of 0.9% NaCl and adding the appropriate volumes of commercial products of KCl, CaCl_2_ dihydrate and sterile water, such that the final solution contains NaCl (8.60 g/L), KCl (0.30 g/L), and CaCl_2_ dihydrate (0.33 g/L). If large volume administration of Ringer's solution is not practical or economic, a clinically acceptable alternative in adult cattle that can swallow and have some forestomach motility is to administer large intravenous volumes of isotonic saline solution (0.9% NaCl) combined with oral administration of KCl boluses (150 g of KCl) and CaCl_2_ boluses (50 g of Ca, equivalent to 111 g of CaCl_2_ or 147 g of CaCl_2_.2H_2_O) (103) or intraruminal administration of similar amounts of KCl and CaCl_2_ in a suitable volume of water.

### Isotonic and Hypertonic Sodium Chloride Solutions

Large volume isotonic saline (0.9% NaCl solution) is clinically inferior to large volume Ringer's solution in treating critically ill adult cattle because 0.9% NaCl solutions have a slightly lower chloride concentration (154 mmol/L) than Ringer's solution (155 mmol/L) and do not include potassium and calcium (9). However, 0.9% NaCl solutions have the advantage of being widely available at low cost in sterile 5 L bags, whereas commercial formulations of Ringer's solution are not available in all countries and must be formulated by the veterinarian.

Following the initial studies of intravenous hypertonic saline in neonatal calves by Constable and colleagues in 1991 (87, 88), the intravenous administration of small volume hypertonic NaCl solutions (7.2%) combined with provision of a large volume OES or water load has been widely adopted for the rapid resuscitation of dehydrated adult cattle (65). The combined treatment protocol provides an inexpensive and practical alternative to the intravenous administration of much large fluid volumes of isotonic saline. Typically, adult cattle are administered 2 or 3 L of hypertonic saline (4–5 ml/kg BW) intravenously over 4–5 min and allowed immediate access to drinking water or an OES (65). The hypertonic saline volume administered to adult cattle is typically 2 or 3 L because most formulations come in 1 L containers; cattle with a body weight range of 400 to 500 kg require 2 L, whereas larger cattle with a body weight range of 600 to 750 kg require 3 L. Intravenous administration of 2 L of hypertonic saline in 5 min can be challenging and requires a 14 g needle and a large pressure head (distance) above the venipuncture site. Intravenous administration of 3 L is very difficult to accomplish within 5 min, and 10–15 min is typically required to administer 3 L in the field without use of an infusion pump. The time taken for administration should be as close to 5 min as possible to obtain the greatest clinical benefit; however, hypertonic saline should not be infused in <5 min because circulatory collapse may occur. The combined treatment of intravenous small volume hypertonic saline and oral large volume water/electrolyte solution rapidly increases plasma volume, increases glomerular filtration rate, and induces a slight metabolic acidosis (65). The volume of hypertonic saline administered should not be increased markedly beyond 4–5 ml/kg BW because infusion of hypertonic saline to euhydrated heifers at 15 ml/kg BW over 15 min resulted in sustained hypernatremia (163 to 170 mmol/L), hyperchloremia (126 to 136 mmol/L) and hyperosmolality (326 to 337 mOsm/kg), without producing any increment in sustained plasma volume expansion (104).

Hypertonic saline is commonly used for the treatment of dairy cattle with endotoxic shock due to coliform mastitis. Small volumes of intravenous hypertonic saline (7.5%, 5 ml/kg BW) increased plasma volume and the cows' voluntary water intake compared to cows treated intravenously with isotonic saline [0.9% NaCl; (105)]. Intravenous hypertonic saline (7.2% NaCl, 2 L over 10 min) has also been administered to cattle with right displaced abomasum, followed by 10 L of 0.9% NaCl IV. The resuscitative effects of this treatment protocol were compared to cattle receiving 26 L of 0.9% NaCl intravenously; the latter volume represented an equivalent sodium load to hypertonic saline treated cows. The initial rate of resuscitation was faster with hypertonic saline compared to conventional large volume fluid administration, based on increases in plasma volume and mean central venous pressure, (106).

Intravenous hypertonic saline (7.5% NaCl, 5 ml/kg over 15 min) was administered to cattle with experimentally induced acute grain overload (ruminal acidosis), and the resuscitative effects compared to that provided by large volume isotonic saline solution (0.9% NaCl). An equivalent response to both fluids was evident, except that there was a slightly larger decrease in jugular venous blood pH and urine pH in cattle treated with hypertonic saline (107, 108). In addition, hypertonic saline treatment increased glomerular filtration and thereby increased the renal excretion of total lactate by 3.5 times that of isotonic saline treatment, resulting in a lower plasma concentration of total lactate (108). It should be noted on theoretical grounds that intravenous hypertonic saline should be less efficacious in ruminants with acute ruminal acidosis. Rumen osmolality is markedly increased in acute ruminal acidosis, decreasing the osmotic gradient that is generated following rapid IV small volume hypertonic saline administration and therefore the free water volume that is osmotically translocated from the forestomach (7).

### Isotonic and Hypertonic Sodium Bicarbonate Solutions

The base excess in cattle with ruminal acidosis that are recumbent or weak and able to stand approximates−20 mmol/L. The estimated bicarbonate deficit in 450 kg body weight cattle exhibiting severe clinicals signs of ruminal acidosis is 3,375 mmol (25 mmol/L × 450 kg BW × 0.3 L/kg BW), assuming an extracellular fluid volume of 30% bodyweight and a base excess in healthy cattle of 5 mmol/L. Intravenous administration of approximately 20 L of isotonic sodium bicarbonate (1.3%, 155 mmol/L) or 3 L of hypertonic sodium bicarbonate (8.4%, 1,000 mmol/L is the preferred initial treatment of ruminal acidosis in severely affected cattle, followed by the administration of oral alkalinizing solutions. Oral rehydration as commonly practiced in dehydrated adult cattle is not suitable for cattle with acute rumen acidosis due to the pre-existing osmotically driven fluid overfill of the rumen that is present in most cases. Specific studies in adult cattle supporting this statement are lacking, but 6% hypertonic sodium bicarbonate solution provided a superior resuscitative response than 7% hypertonic saline in ewes with acute ruminal acidosis (109), particularly related to increased jugular venous blood pH and plasma bicarbonate concentration. Ewes in both groups also underwent rumen lavage and placement of 5 L of water in the rumen (109). A similar response is likely to occur in adult cattle with acute ruminal acidosis.

## Oral Fluid Therapy in Neonatal Calves With Diarrhea

A large number of oral electrolyte solutions are commercially available to treat neonatal calves with diarrhea and dehydration. Most preparations are in the form of powders that are mixed with fresh cow's milk, milk replacer or water, although liquid preparations are available. Oral electrolyte solution (OES) formulations vary but typically they contain electrolytes (sodium, chloride, potassium), alkalinizing agents (bicarbonate or its precursor such as acetate, citrate, and propionate or formate, a metabolizable strong anion), components that facilitate sodium absorption (glucose, glycine, acetate, propionate), and substrates that provide additional energy (glucose, acetate, propionate, glycine). The inclusion of potassium in OES formulations is supported by studies demonstrating that acidemia and dehydration can result in potassium depletion in skeletal muscle of neonatal calves (59), and that potassium depletion in diarrheic calves is associated with the duration of disease and therefore the duration of feed intake depression (60, 62). Some OES formulations contain components to facilitate normalization of the enteric bacterial population (7), such as lecithin-coated pectin fiber that decreases the proliferation of *Escherichia coli* and Salmonella spp (110). Much progress has been made since the 1970's in identifying important attributes of an OES for diarrheic calves. The critical issues in formulating the ideal OES for neonatal calves are **osmolality**, **sodium concentration**, the **effective SID** that reflects the concentration of alkalinizing agents, and the **energy content**. The last three factors are intimately tied to the osmolality of the OES and **the abomasal emptying rate**, and therefore the rate of sodium delivery to the small intestine and ultimately the rate of resuscitation.

Strong ions, both cations (positively charged) and anions (negatively charged), can be categorized as metabolizable or non-metabolizable in biological solutions at physiologic pH. The difference between the charge assigned to all non-metabolizable strong cations (usually sodium and potassium) and non-metabolizable strong anions (usually chloride) in an OES as fed is called the effective SID (111). The alkalinizing ability of an OES therefore reflects the concentration of strong anions such as acetate, propionate, citrate, that are metabolized to bicarbonate, formate that appears to be metabolized to carbon dioxide, and the concentration of the buffer ion bicarbonate, consequently the alkalinizing ability can be quantified by calculating the effective SID of the fed formulation (46, 111, 112).

It should be noted that the ideal OES formulation for neonatal calves with diarrhea differs markedly from the ideal solution for human infants with diarrhea. The primary difference is that neonatal calves have a much lower ability to sweat in response to a high ambient temperature than human infants. Neonatal diarrhea occurs most commonly in human infants in hot climates, and the additional loss of free water and electrolytes in the infant's sweat means that hypernatremia is a common electrolyte abnormality in human infants with diarrhea, whereas hyponatremia is almost always present in neonatal calves with diarrhea that have not received treatment (25, 49, 57). Consequently, OES formulations for human infants typically have lower sodium concentrations (75 mmol/L) and are hypotonic (245 mOsm/kg).

A general recommendation is to routinely administer an OES to all calves < 21 days of age at the first signs of diarrhea, particularly a more liquid diarrhea; this is because it cannot be accurately predicted how quickly the calf will become dehydrated. For diarrheic calves, the total 24-h fluid requirement for rehydration and subsequent maintenance of euhydration is estimated, with the total fluid requirement typically being 12% of euhydrated body weight or greater per day. Ideally, the 24-h fluid requirement should be given orally in at least 2 feedings; preferably at least three separate feedings (two milk and one OES) are provided during the first 24 h of treatment and thereafter in calves with watery diarrhea so that the response to treatment can be monitored.

Intravenous fluids should be administered in addition to an OES in calves that are unable to stand or suckle and in calves that an eye recession of 4 mm or into the orbit (Figure 1). Calves with an eye recession of <4 mm and that are able to suckle and stand can be adequately treated with oral electrolyte solutions alone [Figure 6; (47)], although the rate of resuscitation is slower than that in calves that also received intravenous fluids [Figure 7; (66)].

### Osmolality of the Oral Electrolyte Solution

The osmolality of the OES, as fed, is primarily determined by the sodium and glucose concentrations, and should range from isotonic (300 mOsm/kg) to hypertonic (700 mOsm/kg) when administered to neonatal calves. This range in osmolality is considered optimal because hypotonic solutions (<300 mOsm/kg) have insufficient sodium content to address the hyponatremia and decreased extracellular fluid volume present in diarrheic calves. Hypertonic solutions are considered physiologic when fed as the presence of a countercurrent exchange mechanism at the tip of the intestinal villus results in an effective osmolality of approximately 600 mOsm/kg in healthy milk-fed calves (7). A markedly hypertonic OES should theoretically not be fed to calves with severe villous damage, such as neonatal diarrhea caused by rotavirus or coronavirus; however, we cannot currently predict with sufficient accuracy etiologic agent of calf diarrhea on the basis of the physical examination and measurement of fecal pH (7).

The initial treatment of a dehydrated calf should use an OES that is not added to milk replacer because administering an OES in water provides superior plasma volume expansion (113). It should be noted that low osmolality fluids (300 mOsm/kg) have inadequate energy content because they have insufficient glucose and consequently a low osmolality OES should not be fed to calves when milk is withheld. For this reason, a hypertonic OES (~600 mOsm/kg) is preferred whenever milk is withheld (102, 114). Nevertheless, caution should be applied when administering high osmolality solutions (> 600 mOsm/L) as they decrease abomasal emptying rate (103, 114, 115). This can lead to abomasal bloat and potentially predispose to *Clostridium perfringens* abomasitis. An isotonic OES (~300 mOsm/kg) should be administered whenever milk is concurrently fed because energy dense milk with an osmolality of ~300 mOsm/kg is being provided (110, 115). The OES powder should be added to the milk to provide a solution osmolality of ~600 mOsm/kg, rather than feeding an equivolume mixture of milk and OES, as the latter mixed solution interferes with clot formation in the abomasum and slows the rate of abomasal emptying (116). Ideally, fresh milk should be fed no later than 24 h after the start of treatment to diarrheic calves; fresh cow's milk is preferred to pasteurized waste milk or milk replacer. This is because the energy content of milk is required to maintain body weight, and fresh milk contains trophic factors that facilitate repair of damaged intestinal epithelium (7). In general, milk should not be withheld from diarrheic calves (117), and fresh water must always be provided (118).

### Sodium Concentration of the Oral Electrolyte Solution

The OES should contain a sodium concentration of 90 to 130 mmol/L (117). An important reason to administer an OES is to expand the extracellular volume, and this requires adequate sodium intake. It should be noted that the sodium concentration of cow's milk is relatively low with a mean value of 28 mmol/L in healthy quarters (119, 120), and milk cannot provide the sodium intake required in dehydrated calves with diarrhea. Oral electrolyte solutions with sodium concentrations < 90 mmol/L also provide an inadequate sodium load; conversely, sodium concentrations > 130 mmol/L can result in hypernatremia and additional free water loss (7), particularly if mixed into milk replacer solutions that often have a higher sodium concentration than whole milk (121, 122).

Glucose, acetate, propionate, glycine, and citrate facilitates the absorption of sodium from the gastrointestinal tract lumen and at least one of these substances should be included in the OES. Cotransport mechanisms for sodium with glucose, volatile fatty acids, citrate, and amino acids (such as glycine) in the luminal membrane of villus epithelial cells are unimpaired in calves with enterotoxigenic *E. coli* diarrhea and are thought to be partially functional in malabsorptive/maldigestive diarrheas (7). Glycine was a constituent of the first commercially available OES for calves based primarily on its low cost, wide availability, the likelihood of amino acid coupled sodium transport in the calf's small intestine, and because glycine was included in the first oral rehydration solutions for human infants. The clinical importance of glucose-coupled and glycine-coupled sodium absorption in an OES for diarrheic calves remains uncertain.

The results of a study in euhydrated calves suggested that the importance of glucose-coupled sodium transport in rehydrating calves with diarrhea should be re-evaluated as the ratio of glucose to sodium may not be an important component of treatment efficacy in neonatal calves (123, 124). Additional studies are needed to determine the optimal glucose-to-sodium ratio of an OES for calves; currently the recommended range between 1.0 and 3.0 with an optimum ratio of 1.4 is derived from studies in human infants. It is likely that calves with mild diarrhea and dehydration could be successfully treated with a low sodium hypotonic OES as the total body sodium deficit and anticipated fecal sodium loss are likely to be low (125). In contrast, it is likely the calves with moderate diarrhea and dehydration would benefit from being administered an isotonic OES with a higher sodium concentration, as the total body sodium deficit and anticipated fecal sodium loss are likely to higher (125).

### Effective SID of the Oral Electrolyte Solution

The effective SID of an OES is thought to approximate 40–80 mmol/L because calves with a watery or chronic diarrhea typically have a strong ion (metabolic) acidosis due to the presence of hyponatremia, normochloremia, and varying degrees of hyper D-lactatemia (25, 126, 127). Oral electrolyte solutions with an effective SID = 0 mmol/L create a systemic strong-ion acidosis after being absorbed, and consequently these solutions are not recommended for treating dehydrated calves with diarrhea because they do not contain an alkalinizing agent. Solutions with an effective SID = 0 mmol/L provide an inferior resuscitative response in calves with watery diarrhea to solutions that contain an alkalinizing agent and have an effective SID of at least 40 mmol/L (126). Typical electrolyte and acid-base changes in calves with mild to moderate diarrhea where the fecal characteristics are characterized as soupy have not been well defined, particularly for the first few days of diarrhea in calves with normal appetites for milk.

**Figure 11:**
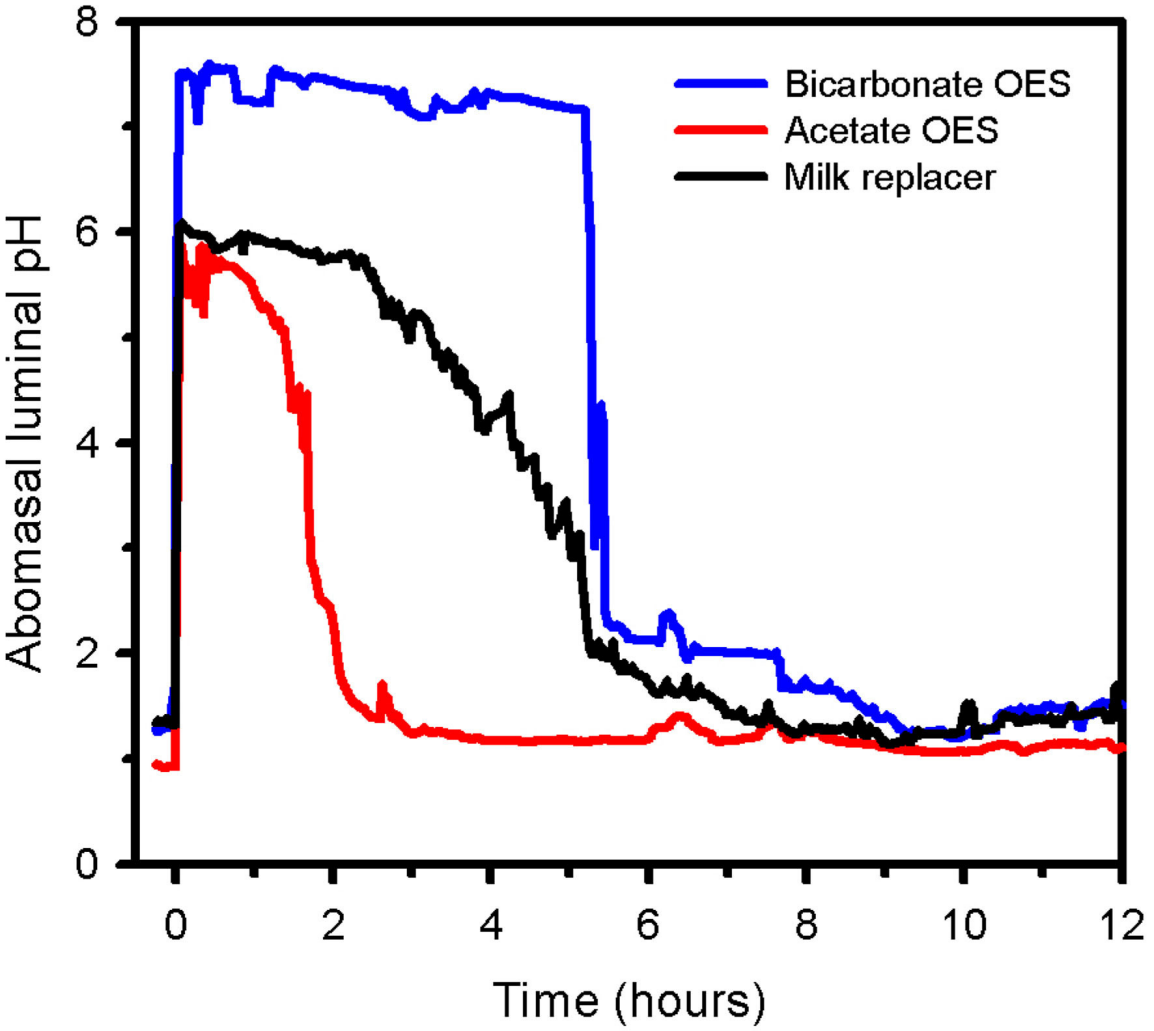
Abomasal luminal pH in a healthy dairy calf suckling an all-milk protein milk replacer (solid black line), a hypertonic high-bicarbonate oral electrolyte solution (OES, blue line), or an isotonic acetate- and propionate-containing OES (red line) in random order at 0 h. Note that the isotonic acetate- and propionate-containing OES is emptied much faster than milk replacer and a hypertonic high-bicarbonate OES. Also note that the hypertonic high-bicarbonate OES causes a marked and sustained increase in abomasal luminal pH. Figure from Peter Constable, unpublished.

The OES for calves with watery diarrhea should contain one or more alkalinizing agents, such as bicarbonate, acetate, propionate, formate, or citrate, at a total concentration range of 40 to 80 mmol/L that reflects an effective SID of 40 to 80 mmol/L (114, 128–131). An effective SID of 60 mmol/L may be optimal (129) and has been adopted by the European Union (130). Bicarbonate-containing solutions are the most effective at rapidly correcting severe acidemia in neonatal calves with diarrhea; however, a theoretical disadvantage of a bicarbonate-containing OES is that they increase abomasal pH and therefore have the potential to decrease the ability to form a milk clot in the abomasum as clotting requires a low abomasal pH [(112); Figure 11]. This theoretical disadvantage does not appear to be true, at least when an OES with bicarbonate concentrations of 25 mmol/L (117), 49 mmol/L (132) or 62 mmol/L (133) are fed in conjunction with cow's milk. A second disadvantage of a bicarbonate containing OES, when fed without milk, is excessive alkalinization of the abomasum (Figure 11), thereby decreasing the effectiveness of low abomasal pH in killing ingested enteric pathogens (9). Of potential clinical significance is that acetate and propionate inhibit growth of Salmonella spp, whereas bicarbonate does not inhibit growth of Salmonella in the intestinal lumen (7).

Acetate, propionate, and citrate must be metabolized from the strong metabolizable anion to bicarbonate to exert an alkalinizing action (117). Acetate and propionate metabolism could be impaired in severely acidemic or dehydrated calves, although this has not been proven. Acetate or propionate can be administered with milk as they do not raise abomasal pH or inhibit the clotting of milk. Citrate complexes Ca^2+^ and consequently inhibit clotting as calcium is needed for milk clotting.

### Rate of Abomasal Emptying

The rate of rehydration in dehydrated calves depends upon the rate that an OES is delivered to the small intestine where fluid is absorbed, and the latter is dependent on the rate of abomasal emptying (7). Interestingly, the abomasal emptying rate is decreased in diarrheic calves when milk or an OES is suckled (133–135). The two most important determinants of emptying rate are the volume and caloric content of an ingested meal (114). The type of protein or fat, osmolality, and duodenal pH also influence abomasal emptying rate, with the rate being decreased when a high OES osmolality is fed or luminal pH is <2.0 or >10.0 (102, 114).

Suckling or esophageal intubation of a high-glucose OES provides a slightly slower rate of abomasal emptying and plasma volume expansion than suckling a low glucose OES (102). Oral electrolyte solutions that provide >2.4 g of glucose/kg BW per day slow the rate of plasma volume expansion and are therefore not recommended for rehydration (102).

### Energy Content of the Oral Electrolyte Solution

**Figure 12:**
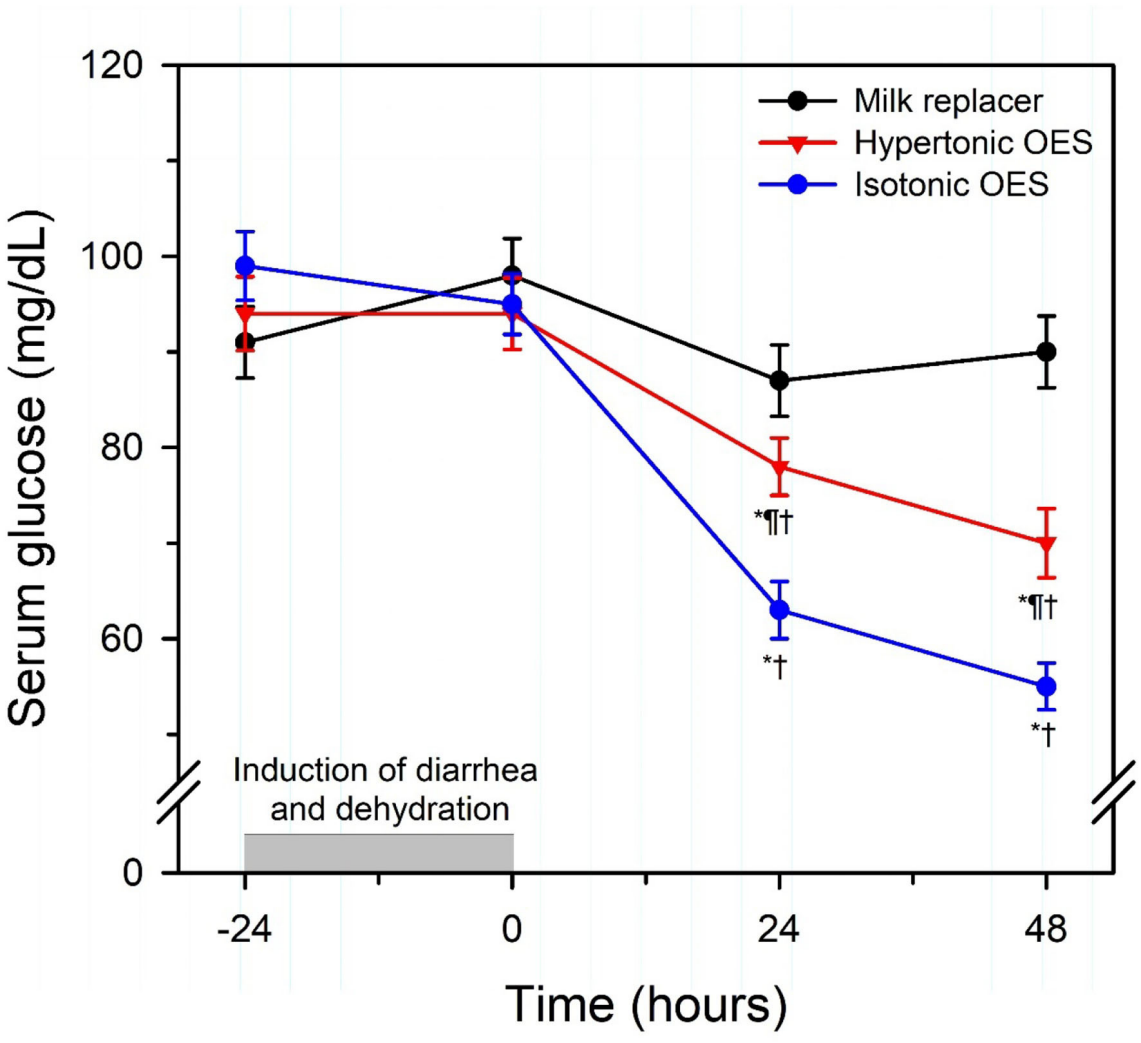
Serum glucose concentrations in neonatal calves with experimentally induced diarrhea and dehydration, and response to oral administration (2 L of solution at 12 h intervals) of milk replacer (*n* = 6), a hypertonic oral electrolyte solution (OES; osmolarity = 606 mOsm/L; glucose = 330 mmol/L; *n* = 6), or an isotonic OES (osmolarity = 307 mOsm/L; glucose = 85 mmol/L; *n* = 6). Note that continued feeding of milk replacer maintained serum glucose concentrations, whereas serum glucose concentration was decreased in calves fed a high glucose or low glucose OES. Values expressed as mean ± SD. *P < 0.05, compared to time = 0 value; ^†^*P* < 0.05, compared to Group M value at the same time interval; ¶*P* < 0.05, compared to Group I value at the same time interval. Reproduced with permission from: Constable et al. (136).

It is now widely accepted that milk should continue to be fed to diarrheic calves while they are receiving an OES, provided that water is readily available to treated calves (118). Historically, milk was withheld from diarrheic calves for 1–2 days and then gradually reintroduced over the next few days. The rationale for milk withholding was the loss of lactase in epithelial cells damaged by common enteropathogens such as rotavirus, coronavirus, and *Cryptosporidium parvum* (7), impairing the calf's ability to digest milk. Feeding an OES alone, even those containing high glucose concentrations, does not provide sufficient energy to the calf and hypoglycemia develops within 8–16 h of feeding the OES and withholding milk [(136), Figure 12]. Feeding milk in addition to an OES shortens the number of days with diarrhea while increasing weight gain and fat stores, and a faster rate of repair of damaged intestinal mucosa than in calves deprived of milk (110).

**Figure 13:**
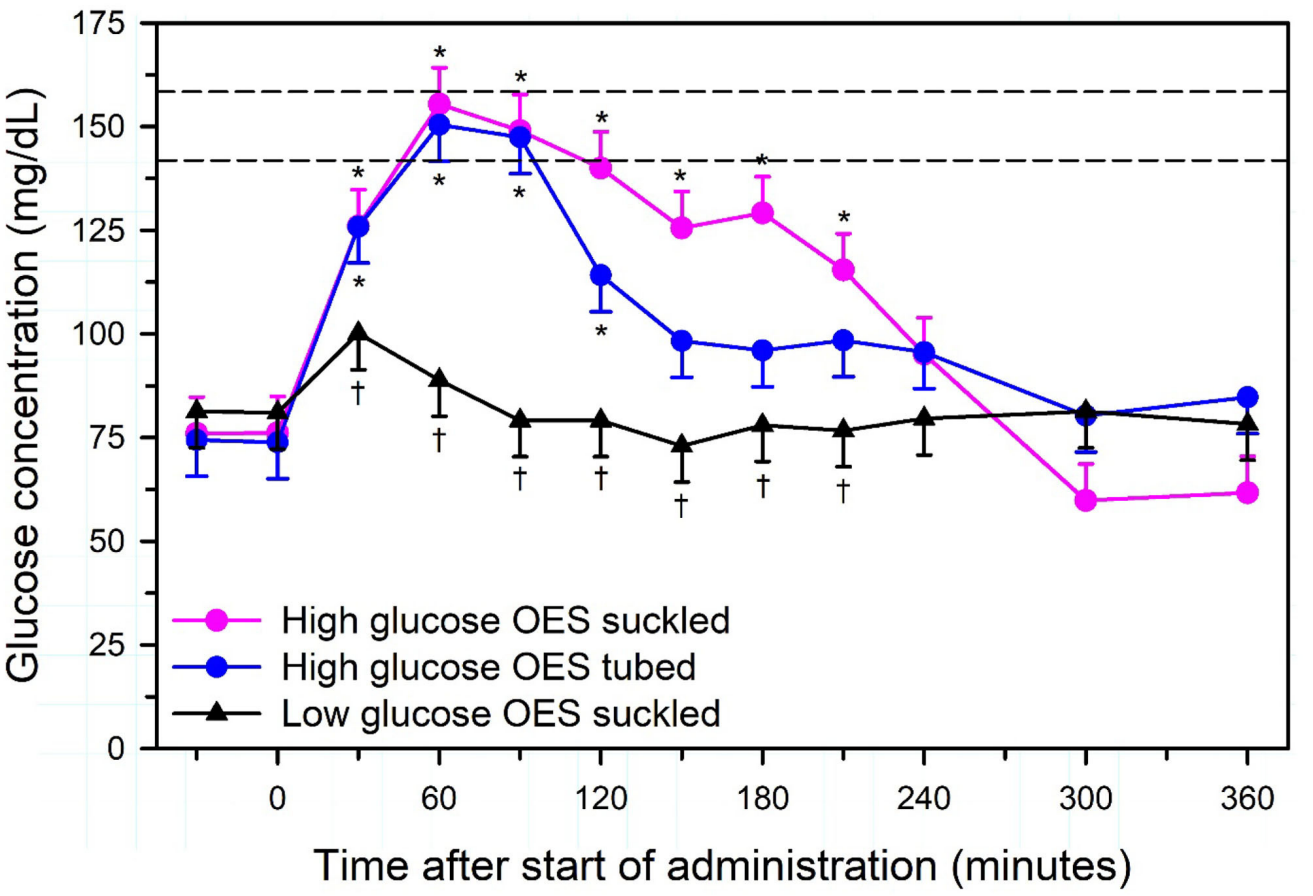
Change in plasma glucose concentration (least squares mean ± standard error) in seven calves that were administered 2 L of a high-glucose oral electrolyte solution (OES; glucose = 405 mmol/L) by suckling or esophageal intubation, or 2 L of a low-glucose OES (glucose = 56 mmol/L) by suckling. The horizontal dashed lines indicate the range of values (140–160 mg/dl) for the renal threshold for glucose in neonatal calves. An asterisk (*) indicates significantly different from the time = 0 min value. A dagger (^†^) indicates significantly different from the value for the high-glucose OES suckled group at the same time. Reproduced with permission from: Nouri and Constable (102).

Moderate to high glucose containing OES should not be administered to diarrheic calves that are also consuming fresh milk or milk replacer as the calves already have adequate energy intake, particularly if the main alkalinizing agent in the OES is propionate. The additional glucose load in these calves may lead to intestinal fermentation if unabsorbed, or glycosuria if hyperglycemia exceeds the renal threshold [140–160 mg/dl] for glucose in neonatal calves [Figure 13, (102)]. This would be accompanied by the additional loss of free water in the feces or urine (121, 122). Whether an OES should contain agents such as glutamine or butyrate that have the potential to assist in repairing damaged intestinal epithelium is being actively researched.

## Oral Fluid Therapy in Adult Cattle

Orally administered sodium is exceptionally well-absorbed in adult cattle (typically oral bioavailability estimates are 90%), and sodium absorption is accompanied by the passive movement of water from the forestomach into the extracellular space (7). Both sodium and water must be absorbed to increase the extracellular fluid volume, with active sodium transport mechanisms in the forestomach accounting for approximately half of the sodium absorbed each day in healthy cattle (9). Maintenance fluid requirements should be administered orally only to adult cattle with normal gastrointestinal motility as oral administration of large fluid quantities to cattle with rumen atony results in ruminal sequestration of fluid and a slower rate of absorption. Although dehydrated cattle with forestomach hypomotility or atony appear to clinically benefit from ororuminal administration of large OES volumes, intravenous fluid administration (including hypertonic saline treatment) is preferred in the initial resuscitation of sick dehydrated cattle with forestomach atony.

The optimal OES formulation for dehydrated cattle is unknown. It is currently believed that an effective OES should contain sodium, potassium, and chloride at a minimum, as dehydrated cattle with decreased feed intake frequently have a hypochloremic, hypokalemic metabolic alkalosis (38). Propionate could be added to the OES as a source of glucose once absorbed and to promote sodium absorption; however, sodium propionate is not as soluble in water as other sodium salts and its inclusion will require a larger administration volume. Calcium, magnesium, and phosphate are often added to the OES in cattle suspected to have mild hypocalcemia, hypomagnesemia, or hypophosphatemia, respectively (9). A variety of oral formulations are commercially available that are dissolved in water and administered via an orogastric tube to adult cattle. These products aim to correct or prevent common electrolyte imbalances of adult cattle but in general are based on empirical evidence while disregarding important aspects of oral rehydration therapy in cattle. Combining salts may provide a convenient supplementation method, but dissolved salts can react with each other, potentially resulting in poorly soluble compounds and a decrease in their oral bioavailability. Such reactions occur when calcium- or magnesium salts are combined with phosphate salts (137–139). Supplemented minerals can furthermore alter the absorption efficiency of other minerals from the digestive tract as they interfere with their transport mechanisms. Such interactions have been described for potassium impairing the transruminal transport mechanisms of magnesium, or sodium reducing the absorption efficiency of potassium from the rumen.

The osmolality of orally administered fluids should be hypotonic if rehydration is an important clinical goal; however, the upper range of tonicity that is safe to administer has not been determined. Estimating the effect an oral drench solution will have on the rumen fluid osmolality requires not only taking into consideration the amount of a salt administered but also the solubility of the salts administered. The solubility of salts in formulations developed for oral administration to adult cattle vary and this is rarely taken into consideration. A net flow of water from rumen to plasma occurs whenever rumen osmolality in adult cattle is > 20 mOsm/kg lower than plasma osmolality, and the rate of water movement from rumen to plasma increases as rumen osmolality decreases (140). Oral electrolyte administration should therefore not produce hyperosmotic rumen fluid, as this will cause free water to move from the extracellular space into the rumen, thereby increasing rumen volume, decreasing the extracellular fluid volume, and increasing the severity of clinical dehydration. Hyperosmotic rumen fluid is the main reason for dehydration in ruminants with ruminal acidosis (grain overload). However, a hypertonic OES requires a lower fluid volume to administer and the added osmolar load should result in additional voluntary water intake by the animal.

One recommended OES formulation for adult cattle, particularly cattle with hypochloremic, hypokalemic, metabolic alkalosis, contains 7 g of NaCl, 1.25 g of KCl, and 0.5 g of CaCl_2_ per L of water, equivalent to 140 g NaCl, 25 g KCl, and 10 g CaCl_2_ in 20 L of water (11). This provides an OES containing 120 mmol/L of NaCl, 16.8 mmol/L of KCl, and 4.5 mmol/L of CaCl_2_, with a calculated osmolarity of 287 mOsm/L. An alternative formulation contains 4 g of NaCl, 1 g of KCl, 2 g of CaCl_2_, and 0.5 g of MgCl_2_ per L of water (141), providing an OES containing 120 mmol/L of Na, 13.4 mmol/L of K, 36 mmol/L of Ca, 11 mmol/L of Mg, and 128 mmol/L of Cl, with a calculated osmolarity of 257 mOsm/L. It should be noted that both formulations contain much less KCl than that recommended (up to 0.4 g of KCl/kg BW for the first 24 h) for treating hypokalemia in adult cattle (142, 143).

Although a minimum volume of 20 L is recommended when administering oral fluids to adult cattle, larger fluid volumes (40 or 60 L) can be safely administered to large adult cattle or cattle with marked dehydration. Rumen volumes in adult cattle typically comprise 13 to 17% of bodyweight (144), equivalent to 104 to 136 L in large cattle (800 kg body weight). In one study, adult beef cattle (420 kg body weight) had water withheld for 3 days while being housed in hot dry conditions; these cattle voluntarily drank 63 L of water in 20 min without any overt clinical signs (145). Ororuminal administration of large volumes of water runs the risk of acute hyponatremia and hypoosmolality due to an osmotically driven translocation of water from the forestomach into the extracellular space. It is therefore preferable to administer hypotonic solutions instead of pure water, and intraruminal administration of 60 L of water containing 250 g of sodium chloride provides a half-isotonic solution with a calculated osmolarity of 142 mOsm/L. It should be noted, however, that ruminants are physiologically adapted to rapidly drink very large water volumes. This is because large water volumes are effectively retained in the rumen due to the increased secretion of hypotonic saliva (145), decreasing the risk of acute hyponatremia. Nevertheless, caution should be exercised when administering large volumes of hypotonic solutions or water to adult cattle, as ororuminal administration of cold solutions could result in cold shock to ruminal microorganisms and hypothermia, and hypoventilation or regurgitation of liquid contents and aspiration pneumonia could occur in cattle with pre-existing ruminal distention.

Oral administration of a sufficient quantity of sodium salts with a high effective SID cause a metabolic alkalosis (strong-ion alkalosis) in adult ruminants, similar to neonatal ruminants. Oral sodium bicarbonate administration provides a useful adjunct treatment in adult ruminants with ruminal acidosis and D-lactate acidosis due to grain overload, as the oral administration of sodium bicarbonate (2.5 g/kg BW) is alkalinizing in adult cattle (146, 147). An important need in fluid and electrolyte therapy for adult ruminants is formulation of a practical, effective, and inexpensive OES.

## Author's Note

The authors have completed a large number of research studies related to intravenous and oral fluid therapy in neonatal calves and adult cattle.

## Author Contributions

All authors listed have been actively engaged in fluid therapy in neonatal calves and adult cattle for at least 10 years and made substantial intellectual contributions to the work. All authors have approved the manuscript for publication.

## Conflict of Interest

The authors declare that the research was conducted in the absence of any commercial or financial relationships that could be construed as a potential conflict of interest.
